# The Impact of Evolving SARS-CoV-2 Mutations and Variants on COVID-19 Vaccines

**DOI:** 10.1128/mbio.02979-21

**Published:** 2022-03-30

**Authors:** Gary McLean, Jeremy Kamil, Benhur Lee, Penny Moore, Thomas F. Schulz, Alexander Muik, Ugur Sahin, Özlem Türeci, Shanti Pather

**Affiliations:** a School of Human Sciences, London Metropolitan University and National Heart and Lung Institute, Imperial College London, London, United Kingdom; b Louisiana State University Health, Shreveport, Louisiana, USA; c Icahn School of Medicine at Mount Sinai, New York, New York, USA; d Centre for HIV and STIs, National Institute for Communicable Diseases of the National Health Laboratory Services, Johannesburg, South Africa; e MRC Antibody Immunity Research Unit, School of Pathology, The University of the Witwatersrand, Johannesburg, South Africa; f Institute of Virology, Hannover Medical School, Hannover, Germany; g Cluster of Excellence 2155 RESIST, Hannover, Germany; h German Centre for Infection Research, Hannover-Braunschweig Site, Germany; i BioNTech, Mainz, Germany; Albert Einstein College of Medicine

**Keywords:** SARS-CoV-2, COVID-19, vaccines, mutation, variant

## Abstract

The emergence of several new variants of severe acute respiratory syndrome coronavirus 2 (SARS-CoV-2) in recent months has raised concerns around the potential impact on ongoing vaccination programs. Data from clinical trials and real-world evidence suggest that current vaccines remain highly effective against the alpha variant (B.1.1.7), while some vaccines have reduced efficacy and effectiveness against symptomatic disease caused by the beta variant (B.1.351) and the delta variant (B.1.617.2); however, effectiveness against severe disease and hospitalization caused by delta remains high. Although data on the effectiveness of the primary regimen against omicron (B.1.1.529) are limited, booster programs using mRNA vaccines have been shown to restore protection against infection and symptomatic disease (regardless of the vaccine used for the primary regimen) and maintain high effectiveness against hospitalization. However, effectiveness against infection and symptomatic disease wanes with time after the booster dose. Studies have demonstrated reductions of varying magnitude in neutralizing activity of vaccine-elicited antibodies against a range of SARS-CoV-2 variants, with the omicron variant in particular exhibiting partial immune escape. However, evidence suggests that T-cell responses are preserved across vaccine platforms, regardless of variant of concern. Nevertheless, various mitigation strategies are under investigation to address the potential for reduced efficacy or effectiveness against current and future SARS-CoV-2 variants, including modification of vaccines for certain variants (including omicron), multivalent vaccine formulations, and different delivery mechanisms.

## INTRODUCTION

Since the first reports of severe acute respiratory syndrome coronavirus 2 (SARS-CoV-2) in humans in December 2019, numerous genetically distinct lineages have evolved (1). Recently, the emergence of several variants carrying mutations with phenotypic implications has raised concerns, as variants with increased transmissibility, disease severity, or ability to escape from antibodies have potential to negatively impact pandemic management strategies. In this article, we review the evolutionary mechanisms underpinning alterations in the genome of SARS-CoV-2 compared with other coronaviruses and RNA viruses; summarize current data on the impact of new variants on authorized mRNA, vector-based, subunit, and inactivated coronavirus disease 2019 (COVID-19) vaccines; and discuss potential mitigation strategies in response to current and future variants of SARS-CoV-2. The search strategy and selection criteria used to identify references for this review are summarized in Text S1 in the supplemental material.

10.1128/mBio.02979-21.4 TEXT S1 Search strategy and selection criteria. Download Text S1, DOCX file, 0.1 MB. Copyright © 2022 McLean et al. 2022 McLean et al. https://creativecommons.org/licenses/by/4.0/ This content is distributed under the terms of the Creative Commons Attribution 4.0 International license.

## SARS-COV-2 EVOLUTIONARY MECHANISMS

Genetic variation in the SARS-CoV-2 genome can arise through two mechanisms: randomly occurring mutations followed by selection, and recombination (2). Random nucleotide sequence errors (substitutions or short deletions/insertions) that occur during replication can alter the amino acid composition of viral proteins (2, 3). In general, RNA viruses accumulate point mutations owing to the low fidelity of the RNA-dependent RNA polymerase (3), but coronaviruses carry a 3′→5′ exoribonuclease that provides proofreading ability, resulting in slower acquisition of mutations (4, 5). Nevertheless, point mutations seem to be major contributors to SARS-CoV-2 evolution (6). The high incidence of mutations detected in the S gene (7), particularly in the receptor-binding domain (RBD) and N-terminal domain (NTD) (8, 9), is likely due to selection for substitutions that improve viral fitness; i.e., through changes in the structure of the spike protein that lead to improved binding to the host receptor or escape from antibody recognition (8, 9). In addition, deletions, which cannot be corrected by the proofreading enzyme (10), are recurrently detected at particular sites in the S gene, primarily located within the NTD, and may contribute to the acquisition of genetic variance by SARS-CoV-2. The increased frequency of these deletions is also likely due to selection for resistance to neutralizing antibodies (10). Rarely, short insertions of a few nucleotides have also been observed (1). Mutations in other genes may also be the result of selection; for example, mutations in the N/Orf9b region have been implicated in enhanced immune escape through suppression of the host innate immune response (11). An unusually high incidence of parallel amino acid substitutions between the RBDs of the spike proteins of SARS-CoV-2- and SARS-CoV-1-related clades suggests the occurrence of evolutionary convergence, possibly as a mechanism of adaptation to the same host cell receptor (12).

RNA recombination occurs at a high rate in coronaviruses (13–16) and has an important role in their evolution. Phylogenetic analyses suggest that recombination events between SARS-CoVs and bat coronaviruses are frequent (17). Although the exact mechanisms by which recombination occurs are unknown, the proofreading exoribonuclease from nonstructural protein 14 (nsp14-ExoN) may be required (18). During replication of coronaviruses, including SARS-CoV-2, a set of subgenomic RNAs is generated, which is thought to increase the homologous recombination rate among closely related genes from different lineages of coronaviruses by template switching (15, 19, 20). Although recombination-mediated changes can theoretically occur at any location, they are detected more frequently in the S gene due to selection favoring changes in the spike glycoprotein (13).

Coronaviruses have the largest known genomes of RNA viruses, allowing for additional plasticity for mutation and recombination relative to viruses with smaller genomes (15). Unlike segmented RNA viruses, such as influenza A, the nonsegmented nature of the coronavirus genome does not allow for evolution via reassortment (3), but conversely, influenza A viruses do not undergo homologous recombination (21). As such, the mutation rate of coronaviruses is considered moderate to high compared with other single-stranded RNA viruses (15). The mutation rate of SARS-CoV-2 has been estimated at a median 1.12 × 10^−3^ mutations per site-year (22), which is similar to those of the related viruses SARS-CoV-1 and Middle East respiratory syndrome coronavirus (MERS-CoV), estimated at 0.80–2.38 × 10^−3^ and 1.12 × 10^−3^ nucleotide substitutions per site-year, respectively (23, 24).

SARS-CoV-2 is assumed to be of zoonotic origin, although the exact zoonotic source of the parental virus and the circumstances behind its emergence in humans in late 2019 remain unknown (25, 26). SARS-CoV-2 shares approximately 96% homology with bat sarbecovirus RaTG13, and specific genes are highly conserved across SARS-CoV-2 and other bat coronaviruses (e.g., SARS-CoV-2 open reading frame 8 [ORF8] shares 94% identity with ZC45 and ZXC21), which suggests a probable bat origin (27, 28). A recent publication reported on bat sarbecoviruses with an RBD of the S protein even more closely related to SARS-CoV-2, one of which could be isolated in human cell cultures, in contrast to RaTG13, for which only a nucleotide sequence is available (29). SARS-CoV-2 has been suggested to be derived from a viral lineage that has been circulating in horseshoe bats for decades (25, 26), and recombination events may have had a role in the origin of the virus (16, 17, 30).

As SARS-CoV-2 circulated globally, the viral genome continued to acquire new mutations, some of which have become widespread. Until late 2020, the most notable was the spike protein mutation D614G. The G614 variant was rare before March 2020, but quickly became dominant, occurring in around three-quarters of all published sequences by June 2020 (31, 32). This rapid spread seems to have been due to increased infectivity, stability, and transmissibility over the ancestral D614 form (32, 33), resulting from a shift to the open configuration of the spike protein trimer, which is required for binding to the host angiotensin-converting enzyme 2 (ACE2) receptor (31) and host cell entry (27).

As of January 25, 2022, 11 global clades of SARS-CoV-2 according to GISAID nomenclature (34) and 25 according to NextStrain nomenclature (35) have been identified. There are several possible contributing factors to the evolution of these different clades. First, the virus is likely still adapting to the new host, as it has been circulating in humans for about 2 years. Second, many countries are experiencing multiple waves of COVID-19 with high infection incidence rates, increasing the probability of advantageous mutations occurring through the sheer number of viral replication events. In addition, as more people recover from SARS-CoV-2 infection or are vaccinated, and population immunity levels increase, selection favors adaptations that evade neutralization by antibodies (36). Increased sequencing coverage in recent months may also have affected the number of variants detected.

Multiple studies have reported long-term shedding of SARS-CoV-2 over several months in immunocompromised individuals (37–41), promoting viral evolution within a single host (37–39, 41). In case reports of immunocompromised patients with COVID-19, treatments such as antivirals (including remdesivir), monoclonal antibody cocktails, or convalescent plasma (37, 39, 41) may have exerted selection pressure, contributing to increased prevalence of antibody escape mutants.

While it is possible that the roll-out of COVID-19 vaccination programs may contribute to viral evolution by increasing selection for immune-escape variants, the reduction in viral circulation resulting from vaccination is expected to result in an overall reduction in the rate of viral adaptation (42).

## SARS-COV-2 VARIANTS

SARS-CoV-2 variants of proven or suspected clinical or epidemiological relevance are designated as a variant of concern (VOC) or variant of interest (VOI) based on criteria such as increased transmissibility and ability to escape immunity (43, 44). Increased transmissibility is of particular concern, as it increases infection rates and can require the introduction of more stringent public health measures. Variant escape from antibody neutralization can reduce the effectiveness of vaccination programs and necessitate the development of modified vaccines or administration of booster doses. Variants with a combination of these characteristics have a significant impact on pandemic management.

Five recently emerged SARS-COV-2 variants have been designated VOCs by the World Health Organization (WHO), as well as other regional agencies (45–47) (Table S1). Notably, all five VOCs exhibit two clusters of S gene mutations—one at the NTD and one at the RBD (1, 48)—both of which are domains targeted by neutralizing antibodies.

10.1128/mBio.02979-21.1 TABLE S1 Characteristics of SARS-CoV-2 variants of concern and variants of interest (1). SARS-CoV-2, severe acute respiratory syndrome coronavirus 2; VOC, variant of concern; VOI, variant of interest. *Exact position and number of mutations may differ according to source; ^†^Excluding D614G; ^‡^Disputed with mutation at same site (2); ^§^Mutations with known or proposed biological significance shown in red. ^¶^The larger group of omicron sequences (B.1.1.529/21M) includes BA.1 (21K) and BA.2 (21L). Data shown here are based on BA.1 as the dominant sequence at the time of writing. Data based on sequences retrieved on 14 September 2021, except for omicron, which was retrieved on 14 January 2022. Download Table S1, DOCX file, 0.06 MB. Copyright © 2022 McLean et al. 2022 McLean et al. https://creativecommons.org/licenses/by/4.0/ This content is distributed under the terms of the Creative Commons Attribution 4.0 International license.

The alpha variant (also known as B.1.1.7, VOC202012/01, or GRY) (1, 46, 49, 50) was initially detected in the United Kingdom in September 2020 (49) and is hypothesized to have emerged from a prolonged infection of an immunocompromised host (51). Alpha has a large number of mutations (27 in total, excluding the now dominant mutation D614G) (1). Of the 27 mutations, 20 (17 nonsynonymous substitutions and three deletions) are amino acid-altering. Eight of these mutations are in the S gene (1) (Table S1), of which four have known biological effects. Mutation N501Y lies within the RBD (49); mutations at this position have previously been shown to affect binding affinity to the ACE2 receptor (52). The deletion at position 69–70 (69/70Δ) has occurred in several other lineages of SARS-CoV-2 in association with RBD changes and may be linked with immune evasion or with infectivity (10, 49, 53). The deletion at position 144 has also been detected in other lineages and may affect the orientation and stability of the spike glycoprotein (54), and confer resistance to NTD-directed neutralizing antibodies (55). Mutation P681H is adjacent to the furin cleavage site at the junction between the S1 and S2 domains of the spike protein and has been shown to promote entry into human lung cells and improve transmissibility in an animal model (49, 56, 57). Recent evidence suggests that the frequency of mutations at position 681 is increasing exponentially worldwide (58).

The beta variant (also known as B.1.351 or GH/501Y.V2) (1, 46, 59, 60) was first detected in South Africa in October 2020 (59). Beta carries 19 mutations (excluding D614G), including eight nonsynonymous mutations in the S gene (1), in addition to variable changes at position L242 (deletion or another nonsynonymous substitution) (59) (Table S1). Three of these mutations are at key sites in the RBD that are associated with immune evasion: N501Y (shared with alpha), E484K, and K417N (59).

The gamma variant (also known as P.1 or GR/501Y.V3) was first detected in Brazil in December 2020 (1, 46, 61). Gamma carries 31 mutations, of which 21 are amino acid-altering (Table S1) (1). These include 20 nonsynonymous substitutions and one deletion (1). Ten mutations affect the spike protein, including two shared with beta (N501Y and E484K), as well as a different mutation at position 417 (K417T) (61).

The delta variant (also known as B.1.617.2 or G/478K.V1) was first documented in India in October 2020 (46). Delta has 21 nonsynonymous mutations, one deletion, and five synonymous mutations (Table S1) (1). Six point mutations affect the spike protein, including P681R (a mutation position shared with alpha and adjacent to the furin cleavage site), and L452R, which is in the RBD and has been linked with increased binding to ACE2 (1, 49, 62) and neutralizing antibody resistance (63). There is also a deletion in the spike protein at position 156/157 (1).

The omicron variant (also known as B.1.1.529 or GRA) was first documented in multiple countries in November 2021 (46). Although it has some mutations in common with the other VOCs, the overall number of mutations is significantly larger than has been seen with any previous variant. Omicron has 45 nonsynonymous mutations, seven deletions, one insertion, and 10 synonymous mutations, with the majority of nonsynonymous mutations located in the S gene at the NTD and RBD (1). Key mutations shared with other VOCs include the deletion at position 69–70 (shared with alpha), K417N (shared with beta), N501Y (shared with alpha), and P681H (shared with alpha) (1). Three subvariants of omicron exist: BA.1 currently dominates in most countries in which omicron is prevalent, but BA.2 seems to have become more common in some countries since January 2022 (64). BA.2 is also referred to as “stealth omicron,” as it lacks the deletion at position 69/70 in the S protein, a mutation characteristic for alpha and BA.1 that is used in mutation-specific PCR assays to differentiate BA.1 from delta. BA.3 is currently still rare (64).

These five VOCs have circulated globally (60, 65) and have become the dominant variants in the geographic regions where they were first identified. As of January 14, 2022, alpha has been reported in 179 countries, beta in 120 countries, gamma in 92 countries, delta in 188 countries, and omicron in 119 countries, each spreading across multiple continents, with omicron currently being the most prevalent VOC in many countries, including the United Kingdom, the United States, and many European countries (65). The rapid spread of alpha, delta, and omicron in particular strongly suggests that these variants have transmission advantages over the ancestral viruses. Based on modeling data, alpha has been estimated to be 43–90% more transmissible than previously circulating variants (66), and delta is thought to be approximately 60% more transmissible than alpha (67). Omicron is highly transmissible, with early estimates suggesting that it may be around 100% more transmissible than delta (68). Some preliminary studies have suggested that omicron is associated with a reduced risk of hospitalization and disease severity compared with delta, although it is not known how much of this is due to increasing population immunity over time (69, 70). Nevertheless, omicron should not be considered to generally cause only mild disease, and its rapid spread is leading to health care systems becoming overwhelmed.

Several other lineages have been classed as VOIs by the WHO, despite not having spread as widely as the five variants described above (46). However, many of these have been reclassified and are no longer being monitored. Currently, only lambda (C.37; GR/452Q.V1) and mu (B1.621; GH) are classed as VOIs, with sporadic transmission (Table S1). Mu has a high accumulation of spike protein mutations seen independently in several other VOIs and VOCs (e.g., E484K, N501Y, and P681H) in addition to the insertion of N at position 146 in the NTD (1, 71). This insertion could potentially affect the S1 closed–open conformation and subsequent binding to ACE2, although the impact on transmissibility and severity of disease is still unknown (71).

**FIG 1:**
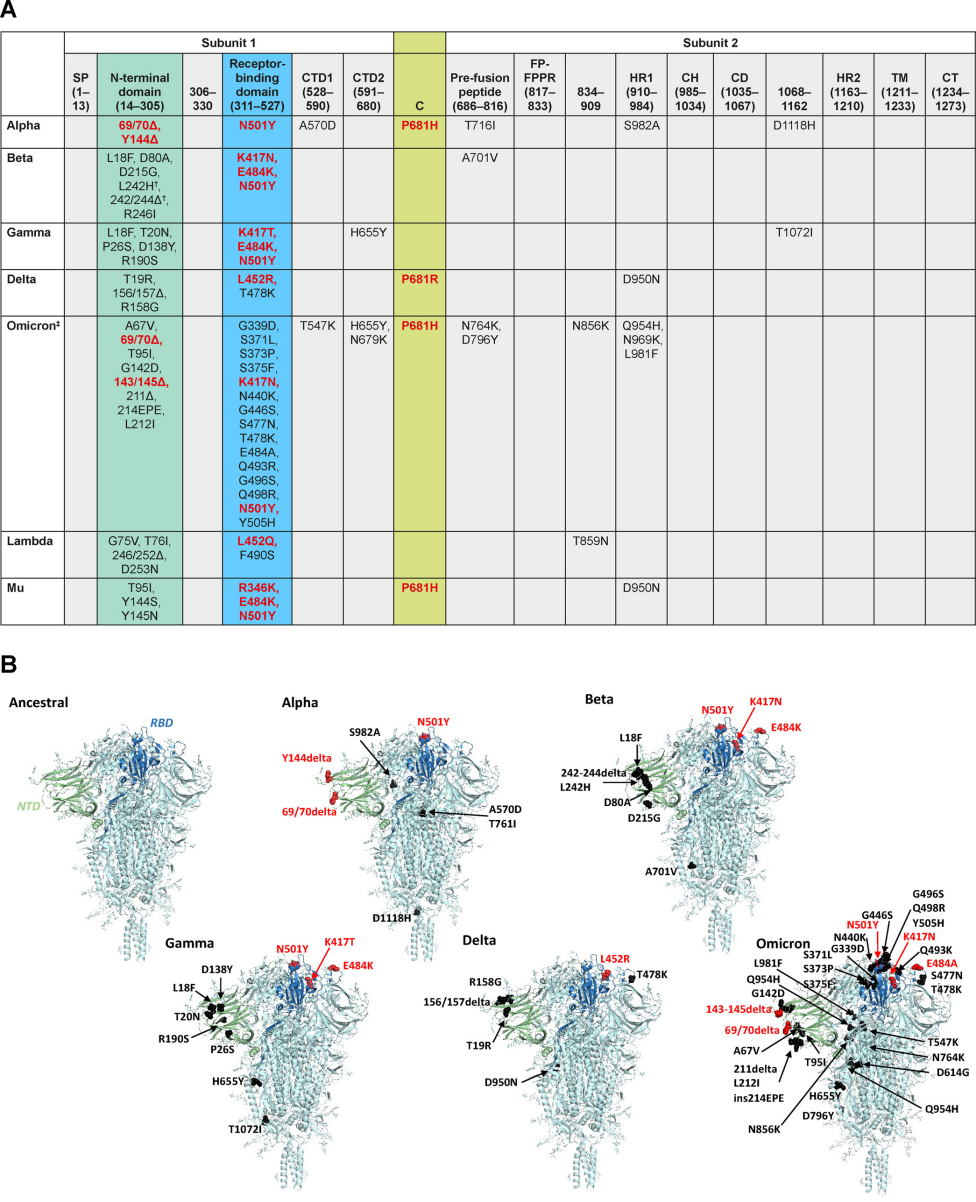
**(**A) Location of spike protein mutations in SARS-CoV-2 variants alpha, beta, gamma, delta, lambda, mu, and omicron (1); (B) 3D structure of the spike protein of SARS-CoV-2 variants alpha, beta, gamma, delta, and omicron in comparison with an ancestral virus created using PyMOL molecular graphic system version 2.3.2 (https://pymol.org). C, cleavage site (residues 681–685); CD, connector domain; CH, central helix; CT, cytoplasmic domain fusion; CTD, C-terminal domain; FP, fusion protein; HR, heptad repeat; SP, signal peptide; TM, transmembrane domain. ^†^Disputed with mutation at same site. ^‡^Based on BA.1 lineage, which is part of the larger group of omicron/B.1.1.529 sequences. Exact position and number of mutations may differ according to source. Structure of spike glycoprotein in A based on Cai et al. (186). Mutations with known or proposed biological significance shown in red. Images in B are based on protein data bank (PDB) entry 6XR8 (https://www.rcsb.org/structure/6xr8). The structure contains D614 but has better solved sequence coverage than other entries although residue P681 is missing and not labeled where mutated in a VOC. Beta: R226I is missing and is not labeled. Gamma: T1072I (in seq is E1072). Omicron: N679K and N969K are missing and not labeled. Domain coloring of spike subunit as follows: NTD: green; RBD: blue; all others including entire subunits 2 and 3: cyan. Mutations are shown in color of function (red: known; black: unknown) and are shown in a single subunit for clarity.

As shown in Fig. 1, mutations in the SARS-CoV-2 spike protein in currently circulating variants are concentrated around the NTD, RBD, and furin cleavage site, suggesting a potential for further mutations to arise in these areas. Although the appearance of the same or similar mutations in multiple variants suggests that they have been driven by evolutionary pressures, the pressures exerted on the virus may change as vaccine programs continue to roll out and new therapeutics are introduced, potentially affecting the specific mutations that will arise in the future.

## IMPACT OF SARS-COV-2 VARIANTS AND MUTATIONS ON IMMUNITY

### *In vitro* studies assessing escape from neutralizing antibodies.

In light of concerns about the potential of new SARS-CoV-2 variants to escape antibodies elicited by vaccination or previous infection, and to resist antibody-based therapeutics (such as monoclonal antibodies or convalescent plasma), numerous studies have evaluated the impact of SARS-CoV-2 variants and mutations on neutralizing antibody activity (55, 72–117). As neutralizing antibody titers represent only one component of the immune response, and correlates of protection are still being established (118, 119), these studies cannot be used to draw conclusions on vaccine efficacy or effectiveness. In addition, as most COVID-19 vaccines elicit very high neutralizing antibody titers (considerably greater than those found in convalescent-phase sera), dramatic fold decreases in neutralizing activity are not necessarily meaningful given the high starting point. Furthermore, titer is not the only indicator of a robust neutralizing antibody response; the nature and quality of antibodies are also important. For example, clonal evolution of SARS-CoV-2 RBD-specific memory B cells over time can result in antibodies with greater resistance to RBD mutations and increased potency (120).

Neutralizing antibody escape studies are valuable for characterization purposes, but comparisons across studies should be made with caution, owing to considerable variations in assay techniques, use of pseudoviruses (of varying construction) versus live virus isolates, vaccine dosing intervals/time since infection, and participant age and immune status, among other factors. In particular, the cell line used to perform neutralization assays can have a considerable effect on results; for example, some studies have used Vero E6 cells, which lack transmembrane protease serine 2 (TMPRSS2), forcing the virus to enter the host cell via the endosomal pathway (121). Antibodies to the NTD of the spike protein are known to be particularly sensitive to pH and may detach in the endosomal environment, leading to an apparent reduction in neutralizing activity. Therefore, findings in Vero E6 cells may be substantially different to those in cell lines that carry TMPRSS2 and, therefore, allow entry at the cell surface.

Nevertheless, some clear patterns have emerged. Reductions in neutralizing activity against new SARS-CoV-2 variants and mutations compared with other circulating lineages have been observed for both convalescent-phase sera and monoclonal antibodies (55, 72, 76, 77, 91–95, 99–103, 105, 108), with beta, omicron, and combinations of mutations including E484K generally resulting in greater reductions than alpha in studies that compared these variants/mutations (55, 72, 77, 92, 102, 109, 114, 115). In addition to beta, E484K may also contribute to antibody escape by gamma and mu, which also carry this mutation (Table S2). Notably, a recent study found that the spike protein of mu escapes neutralization to a degree similar to beta, which had shown the greatest degree of antibody escape prior to the emergence of omicron (122).

10.1128/mBio.02979-21.2 TABLE S2 Studies assessing escape from antibody neutralization by novel variants from sera of COVID-19-vaccinated individuals. Comparisons across these studies should be made with caution, owing to considerable variations in assay techniques, use of pseudoviruses (of varying construction) versus true isolates, vaccine dosing intervals/time since infection, and participant ages and immune status, among other factors. ACE2, angiotensin-converting enzyme 2; CI, confidence interval; GMT, geometric mean titer; IC_50_, half maximal inhibitory concentration; MAb, monoclonal antibody; RBD, receptor-binding domain; SARS-CoV-2, severe acute respiratory syndrome coronavirus 2; TMPRSS2, transmembrane protease serine 2; VSV, vesicular stomatitis virus. Download Table S2, DOCX file, 0.2 MB. Copyright © 2022 McLean et al. 2022 McLean et al. https://creativecommons.org/licenses/by/4.0/ This content is distributed under the terms of the Creative Commons Attribution 4.0 International license.

Studies assessing neutralization of SARS-CoV-2 variants by mRNA vaccine-elicited sera have yielded a similar pattern, although neutralizing activity has generally been retained due to high antibody titers (Table S2). Reductions in neutralizing activity of varying magnitude have been observed against alpha (55, 72, 75–78, 80–83, 85–88, 96, 100, 102), beta (55, 72, 74, 77–79, 84, 85, 87, 88, 96, 100, 102), gamma (72, 79, 85, 89, 90, 102), delta (96, 108), mu (122, 123), and omicron (109–117), when compared with other lineages, with reductions against beta and omicron being notably high (Table S2). Despite the high fold-reductions in neutralization of beta, cell entry was still inhibited at low dilutions (79, 102). Reductions in neutralizing titers have also been observed against viruses carrying the E484K mutation (74, 78, 79, 81, 100, 101, 105), which were generally of greater magnitude than non-E484K mutation combinations (81, 100, 105), suggesting that E484K is a key driver of neutralization escape. Of note, for the lambda variant, which does not carry the E484K mutation, there was no reduction in neutralization relative to that of wild-type SARS-CoV-2 in sera of individuals fully vaccinated with BNT162b2 (124). Reductions in neutralizing titers against variants with mutations at the E484 position (beta, gamma, and mu) were notably smaller or absent in sera from subjects previously exposed to the E484K mutation, suggesting cross-neutralization can occur between variants sharing some or all of the same spike mutations (124). However, reductions in the neutralization of omicron, which carries E484A, have been reported to be greater than with beta in sera from individuals vaccinated with a primary regimen of BNT162b2 (110, 117). A restoration of neutralizing activity against omicron has been reported in sera from individuals who received a booster dose of mRNA vaccine, with even higher titers against delta demonstrated (111, 112, 114–117). Similarly, in a clinical trial of a BNT162b2 primary regimen and booster, the booster dose increased neutralizing antibody titers against the beta variant by 15–20 times compared with post-dose 2, reducing the difference between neutralizing activity against the beta variant and wild-type virus (125).

Fewer studies have assessed escape from vector-based vaccine-elicited antibodies. Reductions in neutralizing activity of post-ChAdOx1 nCoV-19 sera against alpha (85, 86, 104), beta (73), gamma (85), delta (108), and omicron (113) have been observed, with an undetectable neutralization response to beta in eight of 13 samples in one study (73) and to omicron in 20 of 20 samples in another (113). Similarly, in a study of people vaccinated with the vector-based Sputnik V Ad26/Ad5 vaccine (Gamaleya Research Institute of Epidemiology and Microbiology, Moscow, Russian Federation), neutralizing titers against alpha were similar to the D614G control, but 50% of samples did not achieve the IC_80_ threshold for neutralization of beta (106). In a study in which a primary regimen of mRNA vaccines or ChAdOx1 nCoV-19 exhibited reduced neutralizing activity against omicron, sera from all individuals vaccinated with a primary regimen of Sputnik V and all but one vaccinated with a single dose of Ad26.COV2.S had reduced activity against this variant (109). Reduced neutralizing antibody titers against beta and gamma have also been reported in Ad26.COV2.S recipients (98, 126).

One study assessed neutralization activity of sera from people vaccinated with NVX-CoV2373 (a recombinant spike protein subunit vaccine), demonstrating that alpha was neutralized by all samples with modestly diminished activity compared with the control virus (76). In a study assessing neutralization activity of sera from individuals vaccinated with the inactivated vaccine Coronavac, there was no neutralizing activity against omicron after two doses, or after a third CoronaVac dose; however, nine of 10 samples from individuals who received a primary Coronavac regimen and a BNT162b2 booster exhibited neutralizing activity, implying that heterologous boosting with an mRNA vaccine following a vector-based primary regimen results in a broader immune response than a homologous vector-based regimen (112). A primary regimen of the inactivated vaccine BBiBP-CoRV has also been shown to have no neutralizing activity against omicron for the majority of recipients (109). Data on neutralization of other variants and mutations with other vector-based or subunit vaccines are limited.

## POTENTIAL IMPORTANCE OF THE T-CELL RESPONSE

Pharmaceutical research on vaccine-elicited immunity to respiratory viruses tends to focus on the neutralizing antibody response, which is a key element of sterilizing immunity. Neutralizing antibody correlates of protection against symptomatic COVID-19 have been proposed, although evidence of a correlate for asymptomatic infection is lacking (118, 119). However, antibodies represent only one aspect of the immune response to SARS-CoV-2. The T-cell response is important for complete protective immunity, as demonstrated by reports of SARS-CoV-2-exposed individuals with positive T-cell responses but no detectable antibodies (127). Variations in SARS-CoV-2-specific T-cell responses as a function of disease severity have been observed (128, 129), with a coordinated CD4^+^ and CD8^+^ T-cell response associated with milder disease, suggesting a role in protective immunity against COVID-19 (130). Consistent with this, recent studies suggest that the T-cell response may provide protection against SARS-CoV-2 variants. An analysis of T cells from individuals previously infected with SARS-CoV-2 showed that both CD4^+^ and CD8^+^ T cells can recognize multiple epitopes across the SARS-CoV-2 proteome, suggesting that new variants may not easily escape T-cell recognition after natural infection (131). While SARS-CoV-2 may have the capacity to subvert CD8^+^ T-cell surveillance through escape mutations (132), neither alpha nor beta escape CD4^+^ T-cell-mediated vaccine-elicited immunity to the wild-type spike protein (133). In people infected with earlier circulating lineages of SARS-CoV-2, the T-cell response to beta was preserved, despite a loss of CD4^+^ epitope recognition in mutated regions of the spike protein (134).

Vaccines that induce a robust and poly-epitopic cellular response may provide greater protection against novel variants, as a portion of the epitopes recognized by the vaccine-induced T cell repertoire will remain conserved upon further evolution of the virus. Moreover, owing to the highly polymorphic nature of major histocompatibility complex (MHC) class I and II molecules, virus-antigen-specific T-cell responses, in contrast to vaccine-induced antibodies, are not likely to be subject to immune escape on a population level (135, 136).

BNT162b2 has been shown to elicit robust CD8^+^ and CD4^+^ T-cell responses (137), and data from individuals vaccinated with BNT162b2 have shown that the majority of the T-cell response is directed against epitopes conserved across beta, alpha, and the original B lineage (77). Similarly, ChAdOx1 nCoV-19 has been shown to cause expansion of CD4^+^ and CD8^+^ T cells to specific SARS-CoV-2 spike protein epitopes; of 87 epitopes identified, 75 were unaffected by beta mutations (73). A study of cross-recognition of SARS-CoV-2 variants across vaccine platforms, including mRNA-1273, BNT162b2, Ad26.CoV2.S, and NVX-CoV2373, showed preservation of at least 83% and 85% of CD4^+^ and CD8^+^ responses, respectively, regardless of variant (including omicron) (138). In individuals vaccinated with BNT162b2 or Ad26.CoV2.S, the magnitude of T cells with cross-reactivity to omicron was similar to that of beta and delta, despite the significantly larger number of mutations in the omicron variant (139).

## VACCINE EFFICACY AGAINST SARS-COV-2 VARIANTS: EVIDENCE FROM CLINICAL TRIALS

Clinical trials in regions where SARS-CoV-2 variants are prevalent have provided data on COVID-19 vaccine efficacy against those variants (Table S3). A phase 2/3 trial indicated that the efficacy of the ChAdOx1 nCoV-19 vaccine against symptomatic alpha infection is similar to that against previously circulating nonalpha lineages, despite the 9-fold reduction in neutralization activity observed *in vitro* (104). Similarly, the protein-based NVX-CoV2373 vaccine (Novavax) has been shown to have high efficacy (86.3%) against confirmed symptomatic COVID-19 caused by alpha in a clinical trial in the United Kingdom, compared with 95.6% against nonalpha disease (140).

10.1128/mBio.02979-21.3 TABLE S3 Efficacy of COVID-19 vaccines against SARS-CoV-2 variants (1–6). CI, confidence interval; COVID-19, coronavirus disease 2019; HIV, human immunodeficiency virus; PCR, polymerase chain reaction; SARS-CoV-2, severe acute respiratory syndrome coronavirus 2. Download Table S3, DOCX file, 0.08 MB. Copyright © 2022 McLean et al. 2022 McLean et al. https://creativecommons.org/licenses/by/4.0/ This content is distributed under the terms of the Creative Commons Attribution 4.0 International license.

In a phase 1b/2 clinical trial in a limited number of participants without human immunodeficiency virus (HIV) infection in South Africa, the ChAdOx1 nCoV-19 vaccine only provided minimal protection against mild-to-moderate COVID-19 infection from beta (39 cases, vaccine efficacy 10.4%) (73). However, efficacy against severe disease could not be assessed, as the population was low risk (median age 30 years) and the trial was relatively small (73). Efficacy of NVX-CoV2373 in a phase 2 trial in South Africa was 60% in participants without HIV; of 41 COVID-19 events with available sequencing data, 92.7% (38 events) were due to beta (141). Similarly, in the phase 3 ENSEMBLE study, efficacy of a single dose of the Ad26.COV2.S vaccine (Janssen Vaccines & Prevention) against moderate-to-severe COVID-19 was 57% in South Africa, where 95% of COVID-19 events were due to beta (142). Notably, these efficacy values remain above the 50% threshold established by the U.S. Food and Drug Administration (FDA) for COVID-19 vaccine approval (143). In the South African cohort of the phase 3 trial of BNT162b2, in which eight of the nine events were caused by beta and one was of undetermined lineage, efficacy against symptomatic disease was 100% (144).

Efficacy data against the delta variant are limited, as this variant emerged after the primary clinical trials were complete. However, a randomized clinical trial assessing the efficacy of a primary regimen and booster dose of BNT162b2 compared with a primary BNT162b2 regimen and placebo booster has demonstrated vaccine efficacy of 95.6% during a period when delta was the dominant strain (145).

Efficacy data are limited to short duration and early variants, owing to the nature and timing of the clinical trials. Real-world data from ongoing vaccination programs provide further insights into effectiveness against later variants, duration of protection, and need for booster doses.

## VACCINE EFFECTIVENESS AGAINST SARS-COV-2 VARIANTS: EVIDENCE FROM ONGOING VACCINATION PROGRAMS

Ongoing vaccination programs have provided data on COVID-19 vaccine effectiveness against several SARS-CoV-2 variants in a “real-life” setting (Table 1). Data from the mass vaccination campaign in Israel suggest that, consistent with *in vitro* studies, effectiveness of the BNT162b2 mRNA vaccine against alpha is high, with studies carried out during the alpha-dominant period demonstrating vaccine effectiveness after the primary regimen of BNT162b2 up to 94.5% against SARS-CoV-2 infection (146, 147). Similarly, results obtained during the mass vaccination campaign in Scotland also indicate effectiveness against confirmed alpha infections of 92% for BNT162b2 and 73% for ChAdOx1 nCoV-19 after the primary regimen (148). In England, effectiveness of the primary regimen of BNT162b2 or ChAdOx1 nCoV-19 was 93.7% and 74.5%, respectively, against symptomatic COVID-19 (149). Similarly, during the vaccine roll-out in Qatar, the primary regimen of BNT162b2 provided 89.5% protection against confirmed alpha infections and 100% protection against severe, critical, or fatal disease caused by alpha (150). A Canadian study showed that a primary regimen of BNT162b2, mRNA-1273, or ChAdOx1 nCoV-19 was 89%, 91%, and 75% effective against symptomatic infection from alpha (151). Together, these data demonstrate that vaccine effectiveness against alpha after a primary regimen of mRNA- or vector-based vaccines remains high, suggesting that immune escape is unlikely with alpha.

**TABLE 1:** Effectiveness of COVID-19 vaccines against SARS-CoV-2 variants (146, 148–150, 152, 159–163)^*a*^

Variant	Vaccine	Design and setting	Key outcomes	Vaccine effectiveness, % (95% CI)	Reference
Alpha	BNT162b2	Israel; ≥7 days post-dose 2, up to 80% isolates during study period were alpha	PCR-confirmed documented infection	92 (88 to 95)	146
			PCR-confirmed symptomatic COVID-19	94 (87 to 98)	
			Hospitalization due to COVID-19	87 (55 to 100)	
			Severe COVID-19	92 (75 to 100)	
		Israel; ≥7 days post-dose 2, during alpha-dominant period; HCPs	PCR-confirmed infection	94.5 (82.6 to 98.2)	147
			PCR-confirmed symptomatic infection	97.0 (72.0 to 99.7)	
		England; ≥14 days post-dose 2	PCR-confirmed symptomatic COVID-19 caused by alpha^*b*^	93.7 (91.6 to 95.3)	149
		Qatar, ≥14 days post-dose 2	Any PCR-confirmed infection caused by alpha	89.5 (85.9 to 92.3)	150
			Severe, critical, or fatal disease caused by alpha	100 (81.7 to 100)	
		Scotland, ≥14 days post-dose 2	PCR-confirmed infection caused by alpha^*b*^	92 (90 to 93)	148
		Canada, ≥14 days post-dose 2	PCR-confirmed symptomatic infection	89 (87 to 91)	151
	BNT162b2 and/or mRNA-1273 and/or ChAdOx1 nCoV-19^*c*^	Norway, ≥7 days post-dose 2	PCR- or whole genome sequencing-confirmed infection by alpha or delta	84.4 (81.8 to 86.5)	187
	mRNA-1273	Canada, ≥14 days post-dose 2	PCR-confirmed symptomatic infection	91 (84 to 95)	151
	ChAdOx1 nCoV-19	England, ≥14 days post-dose 2	PCR-confirmed symptomatic COVID-19 caused by alpha^*b*^	74.5 (68.4 to 79.4)	149
		Scotland, ≥14 days post-dose 2	PCR-confirmed infection caused by alpha^*b*^	73 (66 to 78)	148
		Canada, ≥14 days post-dose 2	PCR-confirmed symptomatic infection	75 (−98 to 97)	151
Beta	BNT162b2	Qatar, ≥14 days post-dose 2	Any PCR-confirmed infection caused by beta	75.0 (70.5 to 78.9)	150
			Severe, critical, or fatal disease caused by beta	100 (73.7 to 100)	
Delta	BNT162b2	England, ≥14 days post-dose 2	PCR-confirmed symptomatic COVID-19 caused by delta^*b*^	88.0 (85.3 to 90.1)	149
		Scotland, ≥14 days post-dose 2	PCR-confirmed infection caused by delta^*b*^	79 (75 to 82)	148
		UK, ≥14 days post-dose 2, during delta-dominant period	PCR-confirmed infection	80 (77 to 83)	152
		Canada, ≥14 days post-dose 2	PCR-confirmed symptomatic infection	85 (59 to 94)	151
		US, ≥14 days post-dose 2, during delta-dominant period	PCR-confirmed COVID-19 associated hospitalization	80 (73 to 85)	154
			PCR-confirmed COVID-19-associated emergency department/urgent care encounters	77 (74 to 80)	
		US, ≥7 days post-dose 2	PCR-confirmed infection caused by delta	75 (71 to 78)	156
		US, ≥7 days post-dose 2	Hospitalization due to delta	93 (84 to 96)	
		England, 2–≥25 wks post-dose 2 and 1–≥2 wks post-BNT162b2 booster	PCR-confirmed symptomatic disease caused by delta	wks post-dose 2: 2–9: 88.2 (86.7 to 89.5) 10–14: 77.7 (76.3 to 79.0) 15–19: 72.2 (71.0 to 73.4) 20–24: 64.8 (62.6 to 66.9) ≥25: 63.5 (61.4 to 65.5) Wks post-booster: 1–2: 92.2 (90.7 to 93.4) ≥2: 92.6 (92.0 to 93.1)	159
		Denmark, 1–150 days post-dose 2 and 1–30 days post-booster	PCR-confirmed infection caused by delta^*e*^	Days post-dose 2 1–30: 86.7 (84.6 to 88.6) 31–60: 80.9 (79.0 to 82.6) 61–90: 72.8 (71.7 to 73.8) 91–150: 53.8 (52.9 to 54.6) Post-booster: 81.2 (79.2 to 82.9)	161
		England, ≥14 days post-dose 2 and ≥14 days post-mRNA vaccine booster	PCR-confirmed symptomatic infection caused by delta	Cohort analysis: Post-dose 2: 55.9 (55.5 to 56.3) Post-booster: 88.6 (88.1 to 89.1) Test-negative case–control: Post-dose 2: 69.8 (69.4 to 70.2) Post-booster: 94.3 (93.9 to 94.6)	162
	BNT162b2 or mRNA-1273	US, ≥14 days post-dose 2, during delta-dominant period	PCR-confirmed infection	74 (65 to 82)	153
	mRNA-1273	US, ≥14 days post-dose 2 and post-booster	PCR-confirmed infection caused by delta^*d*^	Days post-dose 2: 14–90: 82.8 (69.6 to 90.3) 91–180: 63.6 (51.8 to 72.5) 181–270: 61.4 (56.8 to 65.5) ≥270: 52.9 (43.7 to 60.5) Post-booster: 95.2 (93.4 to 96.4)	160
			Hospitalization caused by delta^*d*^	Post-dose 2: 98.0 (87.2 to 99.7)	
		Denmark, 1–150 days post-dose 2 and 1–30 days post-booster	PCR-confirmed infection caused by delta^*e*^	Days post-dose 2: 1–30: 88.2 (83.1 to 91.8) 31–60: 81.5 (77.7 to 84.6) 61–90: 72.2 (70.4 to 74.0) 91–150: 65.0 (63.6 to 66.3) Post-booster: 82.8 (58.8 to 92.9)	161
	ChAdOx1 nCoV-19	England, ≥14 days post-dose 2	PCR-confirmed symptomatic COVID-19 caused by delta^*b*^	67.0 (61.3 to 71.8)	149
		Scotland, ≥14 days post-dose 2	PCR-confirmed infection caused by delta^*b*^	60 (53 to 66)	148
		UK, ≥14 days post-dose 2, during delta-dominant period	PCR-confirmed infection	67 (62 to 71)	152
		England, 2–≥25 wks post-dose 2 and 1–≥2 wks post-ChAdOx1 nCoV-19 booster	PCR-confirmed symptomatic disease caused by delta	wks post-dose 2: 2–9: 76.2 (63.7 to 84.4) 10–14: 64.9 (55.2 to 72.4) 15–19: 48.5 (44.7 to 52.0) 20–24: 45.4 (43.0 to 47.6) ≥25: 41.8 (39.4 to 44.1) Wks post-booster: 1–2: 87.0 (85.5 to 88.4) ≥2: 93.8 (93.2 to 94.3)	159
		England, ≥14 days post-dose 2 and ≥14 days post-ChAdOx1 nCoV-19 booster	PCR-confirmed symptomatic infection caused by delta	Cohort analysis: Post-dose 2: 25.0 (24.3 to 25.7) Post-booster: 89.7 (88.9 to 90.4) Test-negative case–control: Post-dose 2: 43.7 (43.0 to 44.4) Post-booster: 93.8 (93.3 to 94.3)	162
	BNT162b2 and/or mRNA-1273 and/or ChAdOx1 nCoV-19^*c*^	Norway, ≥7 days post-dose 2	PCR- or whole-genome sequencing-confirmed infection by alpha or delta	64.4 (60.6 to 68.2)	187
	Ad26.COV2.S	US, ≥14 days post-dose 1, during delta-dominant period	PCR-confirmed infection	51 (−2 to 76)	153
		US, ≥14 days post-dose 1, during delta-dominant period	PCR-confirmed COVID-19-associated hospitalization	60 (31 to 77)	154
			PCR-confirmed COVID-19-associated emergency department/urgent care encounters	65 (56 to 72)	
		US, ≥14 days post-dose 2, during delta-dominant period	PCR-confirmed COVID-19-associated hospitalization	95 (92 to 97)	
			PCR-confirmed COVID-19-associated emergency department/urgent care encounters	92 (89 to 93)	
		England, 2–≥25 wks post-dose 2 and ≥2 wks post-BNT162b2 booster	PCR-confirmed symptomatic disease caused by omicron	wks post-dose 2: 2–9: 88.0 (65.9 to 95.8) 10–14: 48.5 (24.3 to 65.0) 15–19: 34.1 (9.7 to 52.0) 20–24: 36.6 (0.4 to 59.6) ≥25: 34.2 (−5.0 to 58.7) Wks post-booster: ≥2: 75.5 (56.1 to 86.3)	159
Omicron	BNT162b2	Denmark, 1–150 days post-dose 2 and 1–30 days post-booster	PCR-confirmed infection caused by omicron^*e*^	Days post-dose 2: 1–30: 55.2 (23.5 to 73.7) 31–60: 16.1 (−20.8 to 41.7) 61–90: 9.8 (−10.0 to 26.1) 91–150: −76.5 (−95.3 to −59.5) Post-booster: 54.6 (30.4 to 70.4)	161
		England, adults aged ≥65 yrs, post-booster	PCR-confirmed symptomatic disease caused by omicron	wks post-BNT162b2 booster: 2–4: 65 5–9: 49 ≥10: 31 Wks post-mRNA-1273 booster: 2–4: 70 5–9: 57	163
	mRNA-1273	US, ≥14 days post-dose 2 and post-booster	PCR-confirmed infection caused by omicron^*d*^	Days post-dose 2: 14–90: 30.4 (5.0 to 49.0) 91–180: 15.2 (0 to 30.7) 181–270: 0 (0 to 1.2) ≥270: 0 (0 to 1.7) Post-booster: 62.5 (56.2 to 67.9)	160
		Denmark, 1–150 days post-dose 2	PCR-confirmed infection caused by omicron^*e*^	Days post-dose 2: 1–30: 36.7 (−69.9 to 76.4) 31–60: 30.0 (−41.3 to 65.4) 61–90: 4.2 (−30.8 to 29.8) 91–150: −39.3 (−61.6 to −20.0)	161
	ChAdOx1 nCoV-19	England, 2–≥25 wks post-dose 2 and 1–≥2 wks post-BNT162b2 booster	PCR-confirmed symptomatic disease caused by omicron	wks post-dose 2: 15–19: −54.7 (−174.0 to 12.6) 20–24: −13.2 (−60.2 to 20.1) ≥25: 5.9 (−29.7 to 31.7) Wks post-booster: 1–2: 71.9 (9.1 to 91.3) ≥2: 71.4 (41.8 to 86.0)	159
		England, adults aged ≥65 yrs, post- BNT162b2 or mRNA-1273 booster	PCR-confirmed symptomatic disease caused by omicron	wks post-booster: 2–4: 62–65 5–9: 48–56 ≥10: 32 (BNT162b2 booster only)	163

a CI, confidence interval; COVID-19, coronavirus disease 2019; HCP, health care professionals; SARS-CoV-2, severe acute respiratory syndrome coronavirus 2.

b Based on presence or absence of deletion in S gene at 69–70.

c ChAdOx1 nCoV-19 was discontinued in Norway on 11 March, 2021, and those who received their first dose were offered a second dose of either BNT162b2 or mRNA-1273. In Norway, a mixed regimen of the two mRNA doses has been administered. The majority of patients in this study (81.3%) received BNT162b2 (187).

d Specimens with S gene target failure were considered to be omicron, and specimens for which S gene was detected were assumed to be delta.

e All specimens investigated for omicron by sequencing or variant-specific PCR; cases not identified as omicron assumed to be delta.

Data on the effectiveness of COVID-19 vaccines against beta and gamma are limited. In Qatar, effectiveness of BNT162b2 against any documented infection with beta was 75%, and effectiveness against severe, critical, or fatal disease caused by beta was 100% (150). In a Canadian study, beta and gamma specimens obtained from patients vaccinated with BNT162b2, mRNA-1273, or ChAdOx1 nCoV-19 were grouped together to evaluate vaccine effectiveness, due to the low number of patients with either variant, as both variants carry N501Y and E484K mutations (151). The primary regimen of BNT162b2 was found to be 85% effective against symptomatic infection and 98% effective against hospitalization or death for this beta/gamma group (151).

The rapid spread of delta has allowed analysis of effectiveness against this variant, with vaccine effectiveness against delta generally remaining high following a primary regimen. In England, vaccine effectiveness against delta was 88.0% with BNT162b2 and 67.0% with ChAdOx1 nCoV-19, with similar findings reported in Scotland (BNT162b2, 79%; ChAdOx1 nCoV-19, 60%) and the United Kingdom as a whole (BNT162b2, 80%; ChAdOx1 nCoV-19, 67%) (148, 149, 152). Similarly, a Canadian study showed that BNT162b2 was 85% effective against symptomatic infection from delta (151). Two doses of BNT162b2 or mRNA-1273 were 74% effective against confirmed infections during a delta-dominant period in the United States, while the one-dose Ad26.COV2.S was 51% effective (153). An interim report from the VISION Network in the United States reported overall effectiveness of 86% for BNT162b2, mRNA-1273, or Ad26.COV2.S against COVID-19-associated hospitalizations during a period when delta accounted for >50% of cases; effectiveness was higher with mRNA-1273 (95%) than with BNT162b2 (80%) and Ad26.COV2.S (60%) (154). However, this interim analysis did not assess effectiveness by time since vaccination; thus, the impact of possible waning of antibody levels is not known. A report from Israel suggested decreased effectiveness of BNT162b2 against COVID-19 infection and symptomatic disease during a period of spread of delta (155); however, this may be an effect of longer intervals post-dose 2 leading to waning antibody levels over time, as a large proportion of the population of Israel was vaccinated in early 2021. The VISION Network observed a significantly lower overall effectiveness of BNT162b2, mRNA-1273, or Ad26.COV2.S against hospitalizations among adults ≥75 years of age compared with adults 18–74 years of age, which may also be a result of waning antibody levels, as adults ≥75 years of age were vaccinated earlier in the United States (154). Similarly, a decline in effectiveness over time since second dose in adults 18–64 years of age was reported in the United Kingdom (152). Furthermore, a study from Southern California found that effectiveness of BNT162b2 against delta infections declined from 93% in the first month after full vaccination to 53% at ≥4 months (156). Similar findings were observed with other SARS-CoV-2 variants, suggesting that reductions in vaccine effectiveness against delta and other variants are likely associated with increased time interval since the primary regimen, rather than vaccine escape (156). Effectiveness of BNT162b2 against severe COVID-19 disease and hospitalization in Israel and in the Southern California study remained high (155, 156). Studies from countries that used wider intervals between the first and second dose of BNT162b2, such as the United Kingdom and Canada, have reported higher vaccine effectiveness against delta infection, although the duration of follow-up has been insufficient to assess the effect of waning (148, 151, 156, 157). This may be due to increased time for proliferation of memory T cells and B cells (120, 158). In more recent studies, in countries where a booster vaccination program has been implemented, vaccine effectiveness of mRNA vaccines and ChAdOx1 nCoV-19 primary regimen with an mRNA booster against delta infection or symptomatic disease has been around 90% or more (159, 160).

Initial data on COVID-19 vaccine effectiveness against the omicron variant suggest that a primary mRNA vaccination regimen provides limited protection against infection. Studies from the United Kingdom, United States, and Denmark have shown that mRNA vaccines have limited-to-moderate effectiveness versus omicron during the first 1–2 months after the second dose (159–162), but with a steep decline in effectiveness thereafter (159, 160). In a study that predicted vaccine effectiveness against omicron relative to delta, controlling for age, region, and ethnic group, effectiveness of two doses of BNT162b2 against omicron was significantly lower than against delta (162). However, in line with neutralizing antibody studies, a booster dose of mRNA vaccine has been shown to partially restore protection (159–162). In England, effectiveness of a primary regimen and booster dose of BNT162b2 against symptomatic omicron disease was 76% (159), and in a test-negative study in the United States, effectiveness of a primary regimen and booster of mRNA-1273 against omicron infection was 63% (160). Reported effectiveness against omicron infection for a primary regimen and booster of BNT162b2 in Denmark was 55% 1 to 30 days after the booster dose (161). Data from the United Kingdom from adults ≥65 years of age who received a primary regimen of BNT162b2 have demonstrated effectiveness against symptomatic omicron of 65% 2 to 4 weeks post-BNT162b2 booster and 70% 2 to 4 weeks post-mRNA-1273 booster (163).

Primary regimens of vector-based vaccines are not effective against omicron (159). In England, effectiveness of two doses of ChAdOx1 nCoV-19 ranged from −55% to 6% from 15 weeks after the second dose (159). However, 2 weeks after a BNT162b2 booster, effectiveness increased to 71% (159).

Despite lower effectiveness against omicron infection, combined vaccine effectiveness against severe disease and hospitalization caused by omicron remains high, with initial data from the United Kingdom reporting effectiveness against hospitalization close to 90% (163, 164). However, preliminary evidence suggests that effectiveness against infection and symptomatic disease wanes with time after the booster dose, with effectiveness of a BNT162b2 primary regimen and booster dropping from 65% at 2–4 weeks post-booster to 49% at 5–9 weeks and 31% at 10 weeks (163, 164). Similar waning was observed with a primary BNT162b2 regimen and mRNA-1273 booster, and a primary ChAdOx1 nCoV-19 regimen with mRNA vaccine booster (163).

## MITIGATION STRATEGIES TO ADDRESS SARS-COV-2 VARIANTS

As described above, data from vaccination programs have highlighted a need for booster doses to increase vaccine effectiveness against certain variants. Therefore, several clinical trials have been conducted to evaluate the safety and immunogenicity of homologous (165–168) and heterologous boosters (169, 170). The randomized trial of a primary regimen and booster of BNT162b2 remains the only booster trial to report efficacy data (145).

In parallel to evaluating booster doses with the original vaccines, the emergence of VOCs led some manufacturers to begin development of variant-specific vaccines in 2021 (165, 171). Since the omicron outbreak, the majority of manufacturers have announced development of omicron-specific vaccines. Currently, BioNTech/Pfizer are exploring beta-, delta-, and omicron-specific BNT162b2 vaccines, as well as a multivalent alpha and delta candidate (172). Initial data suggest that alpha- and delta-specific vaccines, and the multivalent candidate, elicit higher neutralizing activity against omicron than the original vaccine when administered as boosters (173). Moderna is also evaluating delta- and omicron-specific mRNA-1273 vaccines, and is focusing on a multivalent candidate (174). Preliminary analyses show that boosters of modified mRNA-1273 can increase neutralizing antibody titers against beta and gamma (165). AstraZeneca/University of Oxford are reportedly developing modified versions of ChAdOx1 nCoV-19 to target beta and omicron (175, 176), and omicron-specific versions of the Sputnik V Ad26/Ad5 and NVX-CoV2373 vaccines are also in development (177, 178).

Regulatory bodies have issued guidance to vaccine manufacturers on data requirements and processes for approval of modified vaccines (179–182). The WHO Technical Advisory Group on COVID-19 Vaccine Composition (TAG-CO-VAC) has encouraged vaccine developers to gather small-scale data on the breadth and magnitude of immune responses to modified and multivalent vaccines against VOCs (182). Informed by experience with other continuously evolving infectious diseases, such as influenza, approval of modified vaccines will be primarily based on immunogenicity bridging studies to expedite development and review (179–181). Important considerations will include immunogenicity in vaccine-naive and vaccine-experienced individuals, optimal booster dosing, and potential value of multivalent candidates, including both original and modified sequences. An alternative approach to the modification of existing vaccines is the development of new vaccines targeting combinations of viral proteins (e.g., spike, nucleocapsid, and envelope proteins), or with different delivery mechanisms, such as oral or intranasal, to improve the mucosal immune response (183). The TAG-CO-VAC also notes that a pan SARS-CoV-2 vaccine would be a more sustainable long-term option that would effectively be variant-proof (182).

It is worth noting the importance of transmissibility in addition to immune escape features. Beta and omicron both exhibit immune escape, but while the prevalence of beta has remained relatively stable in most regions in recent months, limiting its impact, omicron, which is highly transmissible, has become highly prevalent (65). A risk remains of future emergence of SARS-CoV-2 variants with both increased immune escape and greater replicative and transmission fitness. However, there are no established criteria to indicate whether vaccine adaptation will be required for new variants. Furthermore, this may be dependent on the specific vaccine. For variants without immune escape capability, a vaccine with high efficacy may only require a booster dose to protect against a new variant, while a lower-efficacy vaccine may require adaptation; however, variants with significant immune escape ability are likely to require adaptation for all vaccines. Monitoring of viral mutations by the WHO and international experts, in combination with early warning systems combining structural and computational modeling, will continue to have an essential role in the early identification of high-risk variants as the pandemic continues (46, 184). It is possible that variant-specific vaccines, such as those adapted to beta, delta, or omicron, may elicit cross-reactive neutralizing antibody responses that protect against several different variants (172, 185). T-cell responses induced by each vaccine are also likely to have a role.

## CONCLUSIONS

Current evidence on COVID-19 vaccine performance against SARS-CoV-2 variants is reassuring, demonstrating that alpha has a limited impact on effectiveness and that some vaccine platforms may have the potential to provide at least partial protection against beta and delta infection, likely due to the high levels of neutralizing antibodies elicited and the robust and broad nature of the T-cell response elicited by several of the vaccines. Data from booster vaccination programs suggest that mRNA vaccine boosters can provide some protection against omicron. In addition, vaccine effectiveness against severe disease and hospitalization remains high, suggesting that vaccination has weakened the link between infection and severity of disease. Although vaccine adaptation for existing variants may be unnecessary, strategies are in place to modify vaccines, including different delivery mechanisms, should future emerging variants result in reduced effectiveness. The lower vaccine effectiveness reported against the omicron variant may also be a result of waning immunity over time, underscoring the importance of continuing preventative measures to reduce transmission regardless of vaccination status, as currently recommended by several countries (particularly in the light of the high transmissibility rate of omicron).

Several data gaps remain to be addressed to fully understand the impact of novel variants of SARS-CoV-2 on vaccines. For example, correlates of protective efficacy other than neutralizing antibodies need to be established, the role of other immune effector functions mediated by antibody binding, and the contributions of T-cell responses to protective immunity from vaccination over longer periods of time should be ascertained, to better elucidate the potential risks posed by both existing and future variants.
