# Supplementary material for: The Impact of Evolving SARS-CoV-2 Mutations and Variants on COVID-19 Vaccines
Source: mBio. 2022 Mar 30;13(2):e02979-21. doi: 10.1128/mbio.02979-21 (PMC9040821; doi:10.1128/mbio.02979-21)
Supplement: TEXT S1 [file mbio.02979-21-s0004.docx]

Text S1 Search strategy and selection criteria

References for this review were identified through searches of PubMed and preprint servers medRxiv, bioRxiv, Social Science Research Network (SSRN), and Research Square for English language articles published from December 1, 2019, to January 14, 2022, using the search terms ‘COVID-19’ and ‘SARS-CoV-2’ and other relevant terms for each subtopic, including ‘evolution’, ‘mutation’, ‘variant’, ‘vaccine’, ‘neutralizing antibody’, and ‘convalescent plasma’. Reference lists from articles resulting from these searches were used to identify other relevant articles. Additional sources of information included healthcare agency and SARS-CoV-2 genetic sequence deposition and analysis websites (gisaid.org, covariants.org, and cov-lineages.org). Additional publications were included on a case-by-case basis following the initial literature search owing to the rapidly evolving nature of this topic. Owing to the inclusion of preprint servers in the search, some data included herein had not undergone peer review at the time of writing.
