# Supplementary material for: The Impact of Evolving SARS-CoV-2 Mutations and Variants on COVID-19 Vaccines
Source: mBio. 2022 Mar 30;13(2):e02979-21. doi: 10.1128/mbio.02979-21 (PMC9040821; doi:10.1128/mbio.02979-21)
Supplement: TABLE S1 [file mbio.02979-21-s0001.docx]

Table S1 Characteristics of SARS-CoV-2 variants of concern and variants of interest (1)

| **WHO designation** | **Alpha (VOC)** | **Beta (VOC)** | **Gamma (VOC)** | **Delta (VOC)** | **Omicron (VOC)** | **Lambda (VOI)** | **Mu (VOI)** |
| --- | --- | --- | --- | --- | --- | --- | --- |
| PANGO Lineage | **B.1.1.7** | **B.1.351** | **P.1** | **B.1.617.2** | **B.1.1.529^¶^** | **C.37** | **B.1.621** |
| **Nextstrain clade** | 20I (V1) | 20H (V2) | 20J (V3) | 21A | 21K, 21L, 21M**^¶^** | 21G | 21H |
| **GISAID clade** | GRY | GH/501Y.V2 | GR/501Y.V3 | G/478K.V1 | GRA | GR/452Q.V1 | GH |
| Alternative nomenclature | VOC202012/01 | 501Y.V2 | 501Y.V3 | — | — | — | — |
| Approximate date of detection | September 2020 | October 2020 | December 2020 | October 2020 | November 2021 | December 2020 | January 2021 |
| Number of mutations*^,†^ | 27 (17 non-synonymous substitutions, 3 non-synonymous deletions, 7 synonymous substitutions) | 19 (13 non-synonymous substitutions, 2 non-synonymous deletions, 4 synonymous substitutions) | 31 (20 non-synonymous substitutions,  1 non-synonymous deletion, 10 synonymous substitutions) | 21 (15 non-synonymous substitutions, 1 synonymous deletion, 5 synonymous substitutions/ deletions) | 63 (45 non-synonymous substitutions, 7 deletions, 1 insertion, 10 synonymous substitutions) | 26 (17 non-synonymous substitutions, 2 deletions, 7 synonymous substitutions) | 30 (21 non-synonymous substitutions, 1 non-synonymous deletion, 8 synonymous substitutions) |
| Genes affected*^,†^ | ORF1ab, S, ORF8, N | ORF1ab, S, ORF3a, N, E | ORF1ab, S, ORF3a, ORF8, N | ORF1ab, S, ORF3a, ORF7a,  N, M | ORF1ab, S, N/ORF9b, E, M | ORF1ab, S, N | ORF1ab, S, ORF3a, ORF8, N |

| Amino acid-altering mutations in spike glycoprotein*^,†,§^ | **69/70Δ** **Y144Δ** **N501Y** A570D **P681H** T716I S982A D1118H | L18F D80A D215G L242H^‡^ 242/244Δ^‡^ R246I **K417N E484K N501Y** A701V | L18F T20N P26S D138Y R190S **K417T E484K N501Y** H655Y T1072I | T19R 156/157Δ R158G **L452R** T478K **P681R** D950N | A67V **69/70Δ** T95I G142D **143/145Δ** 211Δ 214EPEins L212I G339D S371L S373P S375F **K417N** N440K G446S S477N T478K E484A Q493R G496S Q498R **N501Y** Y505H T547K H655Y N679K **P681H** N764K D796Y N856K Q954H N969K L981F | G75V T76I 246/252Δ  D253N **L452Q** F490S T859N | T95I  Y144S  Y145N  **R346K**  **E484K**  **N501Y**  **P681H**  D950N |
| --- | --- | --- | --- | --- | --- | --- | --- |

SARS-CoV-2, severe acute respiratory syndrome coronavirus 2; VOC, variant of concern; VOI, variant of interest. *Exact position and number of mutations may differ according to source; ^†^Excluding D614G; ^‡^Disputed with mutation at same site (2); ^§^Mutations with known or proposed biological significance shown in red. ^¶^The larger group of omicron sequences (B.1.1.529/21M) includes BA.1 (21K) and BA.2 (21L). Data shown here are based on BA.1 as the dominant sequence at the time of writing. Data based on sequences retrieved on 14 September 2021, except for omicron, which was retrieved on 14 January 2022.

# References

1. CoVariants. 2022. CoVariants. <https://covariants.org/>. Accessed 14 January, 2022.

2. Tegally H, Wilkinson E, Giovanetti M, Iranzadeh A, Fonseca V, Giandhari J, Doolabh D, Pillay S, San EJ, Msomi N, Mlisana K, von Gottberg A, Walaza S, Allam M, Ismail A, Mohale T, Glass AJ, Engelbrecht S, Van Zyl G, Preiser W, Petruccione F, Sigal A, Hardie D, Marais G, Hsiao NY, Korsman S, Davies MA, Tyers L, Mudau I, York D, Maslo C, Goedhals D, Abrahams S, Laguda-Akingba O, Alisoltani-Dehkordi A, Godzik A, Wibmer CK, Sewell BT, Lourenco J, Alcantara LCJ, Kosakovsky Pond SL, Weaver S, Martin D, Lessells RJ, Bhiman JN, Williamson C, de Oliveira T. 2021. Detection of a SARS-CoV-2 variant of concern in South Africa. Nature 592:438-443.
