# Supplementary material for: The Impact of Evolving SARS-CoV-2 Mutations and Variants on COVID-19 Vaccines
Source: mBio. 2022 Mar 30;13(2):e02979-21. doi: 10.1128/mbio.02979-21 (PMC9040821; doi:10.1128/mbio.02979-21)
Supplement: TABLE S2 [file mbio.02979-21-s0002.docx]

Table S2 Studies assessing escape from antibody neutralization by novel variants from sera of COVID-19-vaccinated individuals

| **Publication** | **Virus types and cell lines** | **Key mutations/variants evaluated** | **Number of samples and timing of collection** | **Key findings** |
| --- | --- | --- | --- | --- |
| **BNT162b2** | | | | |
| Bates T, et al. (1) | Live SARS-CoV-2  Vero E6 cells | Alpha  Beta | *N* = 51  Post-dose 2 (time since dose not stated) | Effective concentration of RBD-binding antibodies was 1.3-fold lower for alpha (*P* = 0.0411) and 1.4-fold for beta (*P* = 0.0047) compared with USA_WA1/2020. Neutralizing antibody titers were reduced by 2.6-fold for alpha and 8.8-fold for beta (both *P* < 0.0001) |
| Cameroni E, et al. (2) | VSV-based pseudovirus  Vero E6 cells  Vero E6-TMPRSS2 cells | Beta  Omicron | *N =* 42  2–4 weeks post-dose 2 | Decreases in neutralization activity of 9-fold against beta and 44-fold against omicron, compared with D614G virus |
| Cele S, et al. (3) | Live SARS-CoV-2  H1299-ACE2Cells  Vero E6 cells | Beta  Omicron | *N = 21*  10–63 days post-dose 2 | 22-fold and 4.3–5.0-fold decreases in neutralizing antibody titers vs ancestral D614G virus for omicron and delta, respectively |
| Chen R, et al. (4) | Live SARS-CoV-2  Vero-TMPRSS2 cells  Vero-hACE2-TMPRSS2 cells | Alpha  Beta S gene  Gamma S gene  Various S gene mutation combinations | *N* = 24  Number of vaccine doses not stated | No reductions in neutralizing activity vs K417N/D614G, moderate reductions vs alpha (2-fold, *P* < 0.01) and E484K/N501Y (4-fold, *P* < 0.0001), large reductions vs beta S gene (10-fold, *P* < 0.0001), compared with WA1/2020 |
| Collier D, et al. (5) | Lentivirus-based pseudovirus  HEK293T-ACE2 cells  HEK293T-TMPRSS2 cells | Alpha | *N* = 21  3 weeks post-dose 2 | Neutralizing antibody titers against alpha reduced by 1.9-fold compared with D614G control |
| Dejnirattisai W, et al. (6) | Live SARS-CoV-2  Vero-81 cells | Alpha  Beta  Gamma | *N* = 25  4–14 days post-dose 2 | Neutralization titers against gamma reduced by 2.6-fold (*P* < 0.0001) compared with Victoria lineage. Neutralization titers against alpha were similar to gamma. Neutralization titers against beta reduced by 7.6-fold |
| Hoffmann M, et al. (7) | VSV-based pseudovirus  African green monkey kidney Vero cells  Vero-TMPRSS2 cells  Human kidney 293T cells  293T-ACE2 cells  Human lung Calu-3 cells  Human colon Caco-2 cells | Alpha S gene  Beta S gene  Gamma S gene | *N* = 15  13–15 days  post-dose 2 | Inhibition of cell entry was slightly reduced for virus with alpha S gene vs wild type. Twelve of 15 samples showed markedly reduced inhibition of pseudo viruses with beta and gamma S genes vs wild type, although all samples completely inhibited entry at the lowest dilution tested |
| Jangra S, et al. (8) | Live SARS-CoV-2  Vero E6 cells | E484K | *N* = 5  Post-dose 2 (47–68 days post-dose 1) | 3.4-fold reduction in neutralization efficiency against E484K compared with USA-WA1/2020 |
| Garcia-Beltran W, et al. (9) | Lentivirus-based pseudovirus  293T-ACE2 cells | Delta  Omicron | <3 months from primary regimen (*N* = 21)  <3 months from booster vaccine (BNT162b2 *N* = 24, mRNA-1273 *N* = 6) | Neutralization of omicron decreased by 122-fold in primary series recipients, with complete loss of neutralization in >50% of individuals; neutralization of delta modestly decreased.  Potent neutralization of omicron in booster recipients (only 4-fold decrease vs wild type). |
| Liu Y, et al. (10) | Live SARS-CoV-2  Cell lines not stated | K417N/E484K/N501Y/D614G  All beta S gene mutations | Post-BNT162b2 sera (*N* = 20)  2 or 4 weeks post-dose 2 (doses 3 weeks apart) | Neutralization of virus with all beta S gene mutations two-thirds weaker vs USA-WA1/2020 (*P* < 0.001). Neutralization of K417N/E484K/N501Y/D614G virus weaker than that of USA-WA1/2020 (*P* = 0.005) and of virus with the dominant D614G substitution |
| Liu Y, et al. (11) | Live SARS-CoV-2  Vero E6 cells | Alpha S gene | *N* = 20  2 or 4 weeks post-dose 2 (doses 3 weeks apart) | Neutralizing titers against virus with alpha gene mutations similar to wild type (597, 469, and 520, respectively) |
| Liu Y, et al. (12) | Live SARS-CoV-2  Vero E6 cells | Delta variant S gene | *N* = 20  2 or 4 weeks post-dose 2 (doses 3 weeks apart) | Geometric mean neutralizing titers were 355 against delta compared with 502 against wild-type virus; however, BNT162b2 sera efficiently neutralized all variants |
| Liu Y, et al. (13) | Live SARS-CoV-2  Vero E6 cells | Lambda S gene | *N* = 20  2 or 4 weeks post-dose 2 (doses 3 weeks apart) | Neutralization of the lambda variant was not reduced relative to wild-type SARS-CoV-2 |
| Messali S, et al. (14) | Live SARS-CoV-2  Cell lines not stated | Mu | *N* = 37  10 and 20 days post-dose 2 (doses 3 weeks apart) | Neutralization of mu was efficient and robust but significantly lower than that observed for parental B.1 |
| Muik A, et al. (15) | VSV-based pseudovirus  Vero76 cells | Alpha S gene | *N* = 40  7–21 days post-dose 2 | Neutralizing titers slightly and significantly reduced but, overall, largely preserved with alpha S gene vs Wuhan reference strain (GMT ratio 0.80 [85% CI 0.71–0.89]) |
| Muik A, et al. (16) | VSV-based pseudovirus  Cell line not stated | Beta  Delta  Omicron | 21 days post-dose 2 (*N* = 32)  1 moth post-booster (*N* = 30) | Neutralization titers 22.8-fold, 6.7-fold, and 2.2-fold lower against omicron, beta, and delta, respectively, compared with Wuhan reference virus after primary regimen  Neutralization titers against omicron increased 23.4-fold after booster dose compared with primary regimen |
| Parry H, et al. (17) | SARS-CoV-2  Vero E6 cells | Gamma | Post-BNT162b2 sera (*N* = 20)  2 weeks post-dose 2 (doses 3 weeks apart) | Neutralizing antibody titers against gamma were reduced by 14-fold compared with the Wuhan/Victoria lineage (*P* = 0.0001), but remained greater than 36 for all samples |
| Peiris M, et al. (18) | Assay type not stated  Cell line not stated | Omicron | 1 month post-dose 2 (*N* = 30)  1 month post-booster (*N* = 23) | 30.9-fold reduction in neutralizing antibody titers against omicron vs wild type after primary regimen  2.8-fold reduction in neutralizing antibody titers against omicron vs wild type after booster dose |
| Planas D, et al. (19) | Live SARS-CoV-2  U2OS-ACE2 GFP1-10 or GFP11 cells | Alpha  Beta | *N* = 19  3 weeks post-dose 2 | Neutralizing antibody titers against beta were 14-fold lower compared with D614G control and 53-fold lower than alpha. 92% of samples neutralized alpha; 77% of the samples neutralized beta with a low titer |
| Planas D, et al. (20) | Vero cells  S-Fuse reporter cells | Alpha  Beta  Delta | *N* = 16  5 weeks post-dose 2 | 16-fold and 3-fold reductions in neutralizing activity of post-BNT162b2 sera were observed against beta and delta, respectively, compared with alpha |
| Rössler A, et al. (21) | Live SARS-CoV-2  Vero cells expressing TMPRSS2 and ACE2 | Alpha  Beta  Delta  Omicron | *N* = 20  1 month post-dose 2 | Neutralizing activity against omicron lower than alpha, beta, and delta. Nine of 20 samples had neutralizing activity versus omicron |
| Skelly DT, et al. (22) | Live SARS-CoV-2  Vero CCL81 cells | Alpha  Beta | *N* = 25  7–17 days post-dose 2 (doses 18–28 days apart) | 2-fold reduction in potency against alpha compared with lineage B, but no case of undetectable neutralization. Greater decline in potency against beta vs lineage B (correlation coefficient 0.74); two samples with undetectable heterotypic neutralization |
| Supasa P, et al. (23) | Live SARS-CoV-2  Vero cells | Alpha | *N* = 25  7–17 days post-dose 2 (doses 3 weeks apart) | Reduction in neutralizing antibody titers against alpha of 3.3-fold (*P* < 0.0001), compared with Victoria lineage |
| Tada T, et al. (24) | Lentivirus-based pseudovirus  293T-ACE2 cells | Alpha S gene mutations (Δ69-70/N501Y/P681H)  N501Y  Δ69–70  Beta S gene mutations | *N* = 5  7 days post-dose 2 (doses 21 days apart) | Reduction in neutralization titers for beta (3.1-fold) and E484K (4.3-fold) compared with D614G wild type. No reduction for N501Y or Δ69-70/N501Y/P681H |
| Uriu K, et al. (25) | Lentivirus-based pseudovirus  HEK293T cells HOS-ACE2/TMPRSS2 cells | Alpha  Beta  Gamma  Delta  Lambda  Mu | *N* = 10  4 weeks post-dose 2 | Mu variant was 7.6-fold more resistant to sera obtained from BNT162b2-vaccinated individuals compared with the parental virus (*P* = 0.0020) |
| Wall E, et al. (26) | Live SARS-CoV-2  Vero E6 cells Vero E6 cells expressing ACE2 and TMPRSS2 | Alpha  Beta  Delta | N = 159  Median 28 days-post-dose 2 | Nine (5%) and six (3%) participants who received two vaccine doses lacked neutralizing activity against beta and delta, respectively. Overall, titers were reduced by 2.6-fold for alpha, 4.9-fold for beta, and 5.8-fold for delta vs wild-type virus |
| Wang P, et al. (27) | Live SARS-CoV-2  Vero E6 cells | Alpha  Beta | *N* = 10, >7 days post-dose 2 (doses 21 days apart) | Neutralizing activity against alpha similar to wild type, but reduced 10.3-fold against beta |
| Wilhelm A, et al. (28) | Live SARS-CoV-2  Caco2 cells | Delta  Omicron | 6 months post-dose 2 *(N* = 15)  0.5 and 3 months post-booster (*N* = 12 and *N* = 20) | 11.4-fold reduction in neutralizing antibody activity against omicron vs delta after primary regimen  37.0-fold and 24.5-fold reductions in neutralization capacity against omicron vs delta 0.5 and 3 months after booster dose |
| Xie X, et al. (29) | Live SARS-CoV-2  Cell line not stated | N501Y | *N* = 20  2–4 weeks post-dose 2 (doses 3 weeks apart) | No reduction in neutralizing activity with Y501 vs N501 |
| Xie X, et al. (30) | Live SARS-CoV-2  Vero E6 cells | Δ69-70/N510Y/D614G  E484K/N501Y/D614G  N501Y | *N* = 20  2–4 weeks post-dose 2 (doses 3 weeks apart) | Neutralization GMT ratio vs the parental virus 1.46 for N501Y, 1.41 for Δ69-70/N501Y/D614G and 0.81 for E484K/N501Y/D614G |
| **mRNA-1273** | | | | |
| Cameroni E, et al. (2) | VSV-based pseudovirus  Vero E6 cells  Vero E6-TMPRSS2 cells | Beta  Omicron | *N* = 34  2–4 weeks post-dose 2 | Decreases in neutralization activity of 10-fold against beta and 33-fold against omicron, compared with D614G virus |
| Doria-Rose N, et al (31) | Lentivirus-based pseudovirus  Cell lines not stated | Beta  Omicron | 4 weeks post-dose 2 (*N* = 30)  2 weeks post-booster (*N* = 7) | Neutralizing antibody titers 41- to 84-fold lower against omicron and 9.2- to 13.6-fold lower against beta vs ancestral D614G virus after primary regimen  Neutralizing antibody titers 4.2- to 6.5-fold lower against omicron and 2.6- to 3.4-fold lower against beta vs ancestral D614G virus after booster |
| Edara V, et al. (32) | Live SARS-CoV-2  Vero cells | Beta | *N* = 19  14 days post-dose 2 | 3.8-fold reduction in neutralizing antibody titer for beta compared with lineage B.1 (*P* < 0.0001); however, all samples retained neutralization capacity |
| Edara V, et al. (33) | Live SARS-CoV-2  Vero E6 cells | Alpha  N501Y | *N* = 14  14 days post-dose 2 | Neutralizing antibody titers were significantly lower against alpha (*P* = 0.02) and N501Y (*P* = 0.02) compared with the A.1. lineage, but similar to the B.1 lineage |
| Garcia-Beltran W, et al. (9) | Lentivirus-based pseudovirus  293T-ACE2 cells | Delta  Omicron | <3 months from primary series (*N* = 24)  <3 months from booster vaccine (mRNA-1273 *N* = 32, BNT162b2 *N* = 1) | Neutralization of omicron decreased by 43-fold in primary regimen recipients, with complete loss of neutralization in >50% of individuals; neutralization of delta modestly decreased  Potent neutralization of omicron in booster recipients (only 6-fold decrease vs wild type) |
| Rössler A, et al. (21) | Live SARS-CoV-2  Vero cells expressing TMPRSS2 and ACE2 | Alpha  Beta  Delta  Omicron | *N* = 10  4–6 months post-dose 2 | Neutralizing activity against omicron lower than alpha, beta, and delta. One of 10 samples had neutralizing activity versus omicron |
| Shen X, et al. (34) | Lentivirus-based pseudovirus  293T-ACE2.MF cells | Alpha | *N* = 40  11 samples 28 days post-dose 1, 29 samples 28 days post-dose 2 | Alpha neutralized, although with modestly diminished susceptibility compared with a D614G variant (median IC_50_ on average 2.1-fold lower) |
| Wang P, et al. (27) | Live SARS-CoV-2  Vero E6 cells | Alpha  Beta | *N* = 12  15 days post-dose 2 (doses 28 days apart) | Neutralizing activity against alpha similar to wild type, but reduced 12.4-fold against beta |
| Wilhelm A, et al. (28) | Live SARS-CoV-2  Caco2 cells | Delta  Omicron | 6 months post-dose 2 *(N* = 14)  0.5 months post-BNT162b2 booster (*N* = 9) | 20-fold reduction in neutralizing antibody activity against omicron vs delta after primary regimen  22.7-fold reductions in neutralization capacity against omicron vs delta post-booster |
| Wu K, et al. (35) | VSV-based pseudovirus  Cell lines not stated | Beta spike protein  Gamma spike protein  Alpha spike protein/E484K | *N* = 8  1 week post-dose 2 | Decrease in titer of neutralizing antibodies against beta, gamma and alpha/E484K; largest reduction (factor of 6.4) was against beta; however, all sera fully neutralized beta at relatively low dilutions |
| Wu K, et al. (36) | VSV and lentivirus-based pseudo viruses  293T-hACE2.mF cells  A549-hACE2-TMPRSS2 cells | Alpha  Beta  K417N/E484K/N501Y/ D614G | *N* = 8  1 week post-dose 2 (36 days post-dose 1) | No significant impact on neutralization of alpha; 6.4-fold and 2.7-fold reduction in neutralization of beta and K417N/E484K/N501Y/D614G, respectively, compared with D614G pseudovirus |
| **mRNA vaccines combined** | | | | |
| Schmidt F, et al. (37) | HIV-1-based pseudovirus  HT1080/ACE2.cl14 cells | Omicron | 1.3 months post-dose 2 (*N* = 18)  1 month post-booster (*N* = 18) | 127-fold loss of potency against omicron vs Wuhan-hu-1 virus  18-fold loss of potency against omicron vs Wuhan-hu-1 virus |
| Wang P, et al. (38) | VSV-based pseudovirus and live SARS-CoV-2  Vero E6 cells | Gamma | mRNA-1273: *N* = 12, 15 days post-dose 2 (doses 28 days apart)  BNT162b2: *N* = 10, >7 days post-dose 2 (doses 21 days apart) | Reduction of neutralizing activity in every sample, but magnitude was modest (2.2–2.8-fold for pseudovirus; 3.8–4.8-fold for authentic SARS-CoV-2 P.1) |
| Wang Z, et al. (39) | HIV-1-based pseudovirus  VSV-based pseudovirus  HT1080ACE2.cl14 cells  293T-ACE2 cells  293T-ACE2.cI12 cells | K417N/E484K/N501Y  E484K  K417N  N501Y | mRNA-1273: *N* = 14, post-dose 2  BNT162b2: *N* = 6, post-dose 2 | 1–3-fold decrease in neutralizing activity against E484K, N501Y, and K417N/E484K/N501Y combination. Of 17 of the most potent mAbs isolated from vaccine sera, 9, 5, and 4 were ≥10-fold less effective against E484K, K417N, and N501Y, respectively |
| **ChAdOx1-nCoV-19** | | | | |
| Cameroni E, et al. (2) | VSV-based pseudovirus  Vero E6 cells  Vero E6-TMPRSS2 cells | Beta  Omicron | 2–4 weeks post-dose 2 (*N =* 12*)* | Decreases in neutralization activity of 12-fold against beta and 36-fold against omicron, compared with D614G virus |
| Dejnirattisai W, et al. (6) | Live SARS-CoV-2  Vero-81 cells | Alpha  Beta  Gamma | *N* = 25  4–14 days post-dose 2 | Neutralization titers against gamma reduced by 2.9-fold (*P* < 0.0001) compared with Victoria lineage. Neutralization titers against alpha were similar to gamma. Neutralization titers against beta reduced by 9-fold |
| Emary K, et al. (40) | Live SARS-CoV-2  Vero E6 cells | Alpha  Beta | *N* = 49  Vaccine dose not stated | Neutralization activity 9-fold lower against alpha compared with non-alpha lineage |
| Madhi S, et al. (41) | Lentivirus-based pseudovirus and live SARS-CoV-2  293T-ACE2.MF cells (pseudovirus assay) and Vero E6 cells (live assay) | Beta  K417N/E484K/N501Y | *N* = 13  2 weeks post-dose 2 (doses 21–35 days apart) | Neutralization was lower with live-virus vs pseudovirus assay. In the live virus assay, eight serum samples had undetectable neutralization response to beta; the remaining five showed a 4.1–31.5-fold reduction in activity |
| Planas D, et al. (20) | Vero cells  S-Fuse reporter cells | Alpha  Beta  Delta | *N* = 20  4 weeks post-dose 2 | Compared with alpha, there was a 5-fold reduction in neutralization titers against delta and a 9-fold reduction against beta |
| Rössler A, et al. (21) | Live SARS-CoV-2  Vero cells expressing TMPRSS2 and ACE2 | Alpha  Beta  Delta  Omicron | 1 month post-dose 2 (*N* = 10) | No samples had neutralizing activity versus omicron |
| Supasa P, et al. (23) | Live SARS-CoV-2  Vero cells | Alpha | *N* = 25  14–28 days post-dose 2 | Reduction in neutralizing antibody titers against alpha of 2.1–2.5-fold (*P* < 0.002) compared with Victoria lineage |
| **NVX-CoV2373** | | | | |
| Shen X, et al. (34) | Lentivirus-based pseudovirus  293T-ACE2.MF cells | Alpha | *N* = 28  2 weeks post-dose 2 | Alpha neutralized, although with modestly diminished susceptibility compared with a D614G variant (median IC_50_ on average 2.1-fold lower) |
| **Ad26.COV2.S** | | | | |
| Alter G, et al (42) | Live SARS-CoV-2 and lentivirus-based pseudovirus  Vero E6 USAMRID cells and HEK293T-hACE2 cells | Alpha  Beta  Gamma | *N* = 20  71 days post-dose1 (single dose cohorts) or 14 days post-dose 2 (two dose cohorts) | Fold reductions in neutralizing activity were 5.0 vs beta and 3.3 vs gamma in the pseudovirus assay, and 10.6-fold vs beta in the live SARS-CoV-2 assay, when compared with WA1/2020 |
| Cameroni E, et al. (2) | VSV-based pseudovirus  Vero E6 cells  Vero E6-TMPRSS2 cells | Omicron | *N* = 12  1–19 weeks post-dose 1 | Only one of 12 samples exhibited neutralization activity against omicron |
| Garcia-Beltran W, et al. (9) | Lentivirus-based pseudovirus  293T-ACE2 cells | Delta  Omicron | <3 months from primary series (*N* = 20)  <3 months from booster vaccine (Ad26.COV2.S *N* = 1; mRNA-1273 N = 7) | Neutralization of omicron and delta decreased in primary regimen recipients  Potent neutralization of omicron in booster recipients (only 13-fold decrease vs wild type) |
| Moore P, et al (43) | Lentivirus-based pseudovirus  HEK293T cells overexpressing ACE2 | Beta S gene | *N* = 27  29 days post-dose 1 | 82% of sera (n=22) showed no detectable neutralization of beta. Geometric mean titer reduced 3-fold from 152 to 48 |
| Schmidt F, et al. (37) | HIV-1-based pseudovirus  HT1080/ACE2.cl14 cells | Omicron | *N* = 19  1 month post-dose 1 | Majority of samples did not contain detectable neutralizing activity against omicron post-dose 1 |
| **Sputnik V Ad26/Ad5** | | | | |
| Cameroni E, et al. (2) | VSV-based pseudovirus  Vero E6 cells  Vero E6-TMPRSS2 cells | Omicron | *N* = 11  2–4 weeks post-dose 2 | 0 of 11 samples exhibited neutralization activity against omicron |
| Ikegame S, et al. (44) | VSV-based pseudovirus  293T-ACE2-TMPRSS2 cells | Alpha S gene  Beta S gene | *N* = 12  1 month post-dose 2 | 50% of samples failed to achieve IC_80_ against virus with beta S gene mutations at highest concentration tested (1:20); titers were 6.8-fold weaker compared with virus with G614 control S gene |
| **Coronavac** | | | | |
| Peiris M, et al. (18) | Assay type not stated  Cell line not stated | Omicron | 1 month post-dose 2 (*N* = 30)  1 month post-Coronavac booster (*N* = 10)  1 month post-BNT162b2 booster (*N* = 10) | 0 of 30 and 0 of 10 samples after primary regimen and Coronavac booster exhibited neutralizing activity against omicron  Nine of 10 samples post-BNT162b2 booster exhibited neutralizing activity against omicron |
| **BBiBP-CORv** | | | | |
| Cameroni E, et al. (2) | VSV-based pseudovirus  Vero E6 cells  Vero E6-TMPRSS2 cells | Omicron | *N* = 13  2–4 weeks post-dose 2 | Only three of 13 samples exhibited neutralization activity against omicron |

Comparisons across these studies should be made with caution, owing to considerable variations in assay techniques, use of pseudoviruses (of varying construction) versus true isolates, vaccine dosing intervals/time since infection, and participant ages and immune status, among other factors. ACE2, angiotensin-converting enzyme 2; CI, confidence interval; GMT, geometric mean titer; IC_50_, half maximal inhibitory concentration; mAb, monoclonal antibody; RBD, receptor-binding domain; SARS-CoV-2, severe acute respiratory syndrome coronavirus 2; TMPRSS2, transmembrane protease serine 2; VSV, vesicular stomatitis virus.

# References

1. Bates TA, Leier HC, Lyski ZL, McBride SK, Coulter FJ, Weinstein JB, Goodman JR, Lu Z, Siegel SAR, Sullivan P, Strnad M, Brunton AE, Lee DX, Curlin ME, Messer WB, Tafesse FG. 2021. Neutralization of SARS-CoV-2 variants by convalescent and vaccinated serum. medRxiv doi:10.1101/2021.04.04.21254881:2021.04.04.21254881.

2. Cameroni E, Saliba C, Bowen JE, Rosen LE, Culap K, Pinto D, VanBlargan LA, De Marco A, Zepeda SK, Iulio Jd, Zatta F, Kaiser H, Noack J, Farhat N, Czudnochowski N, Havenar-Daughton C, Sprouse KR, Dillen JR, Powell AE, Chen A, Maher C, Yin L, Sun D, Soriaga L, Bassi J, Silacci-Fregni C, Gustafsson C, Franko NM, Logue J, Iqbal NT, Mazzitelli I, Geffner J, Grifantini R, Chu H, Gori A, Riva A, Giannini O, Ceschi A, Ferrari P, Cippà P, Franzetti-Pellanda A, Garzoni C, Halfmann PJ, Kawaoka Y, Hebner C, Purcell LA, Piccoli L, Pizzuto MS, Walls AC, Diamond MS, Telenti A, Virgin HW, Lanzavecchia A, Veesler D, Snell G, Corti D. 2021. Broadly neutralizing antibodies overcome SARS-CoV-2 Omicron antigenic shift. bioRxiv doi:10.1101/2021.12.12.472269:2021.12.12.472269.

3. Cele S, Jackson L, Khoury DS, Khan K, Moyo-Gwete T, Tegally H, San JE, Cromer D, Scheepers C, Amoako D, Karim F, Bernstein M, Lustig G, Archary D, Smith M, Ganga Y, Jule Z, Reedoy K, Hwa S-H, Giandhari J, Blackburn JM, Gosnell BI, Karim SSA, Hanekom W, NGS-SA, Team C-K, von Gottberg A, Bhiman J, Lessells RJ, Moosa M-YS, Davenport MP, de Oliveira T, Moore PL, Sigal A. 2021. SARS-CoV-2 Omicron has extensive but incomplete escape of Pfizer BNT162b2 elicited neutralization and requires ACE2 for infection. medRxiv doi:10.1101/2021.12.08.21267417:2021.12.08.21267417.

4. Chen RE, Zhang X, Case JB, Winkler ES, Liu Y, VanBlargan LA, Liu J, Errico JM, Xie X, Suryadevara N, Gilchuk P, Zost SJ, Tahan S, Droit L, Turner JS, Kim W, Schmitz AJ, Thapa M, Wang D, Boon ACM, Presti RM, O'Halloran JA, Kim AHJ, Deepak P, Pinto D, Fremont DH, Crowe JE, Jr., Corti D, Virgin HW, Ellebedy AH, Shi PY, Diamond MS. 2021. Resistance of SARS-CoV-2 variants to neutralization by monoclonal and serum-derived polyclonal antibodies. Nat Med 27:717-726.

5. Collier DA, De Marco A, Ferreira I, Meng B, Datir RP, Walls AC, Kemp SA, Bassi J, Pinto D, Silacci-Fregni C, Bianchi S, Tortorici MA, Bowen J, Culap K, Jaconi S, Cameroni E, Snell G, Pizzuto MS, Pellanda AF, Garzoni C, Riva A, Collaboration C-NBC-, Elmer A, Kingston N, Graves B, McCoy LE, Smith KGC, Bradley JR, Temperton N, Ceron-Gutierrez L, Barcenas-Morales G, Consortium C-GU, Harvey W, Virgin HW, Lanzavecchia A, Piccoli L, Doffinger R, Wills M, Veesler D, Corti D, Gupta RK. 2021. Sensitivity of SARS-CoV-2 B.1.1.7 to mRNA vaccine-elicited antibodies. Nature 593:136-141.

6. Dejnirattisai W, Zhou D, Supasa P, Liu C, Mentzer AJ, Ginn HM, Zhao Y, Duyvesteyn HME, Tuekprakhon A, Nutalai R, Wang B, Lopez-Camacho C, Slon-Campos J, Walter TS, Skelly D, Costa Clemens SA, Naveca FG, Nascimento V, Nascimento F, Fernandes da Costa C, Resende PC, Pauvolid-Correa A, Siqueira MM, Dold C, Levin R, Dong T, Pollard AJ, Knight JC, Crook D, Lambe T, Clutterbuck E, Bibi S, Flaxman A, Bittaye M, Belij-Rammerstorfer S, Gilbert SC, Carroll MW, Klenerman P, Barnes E, Dunachie SJ, Paterson NG, Williams MA, Hall DR, Hulswit RJG, Bowden TA, Fry EE, Mongkolsapaya J, Ren J, Stuart DI, Screaton GR. 2021. Antibody evasion by the P.1 strain of SARS-CoV-2. Cell 184:2939-2954 e9.

7. Hoffmann M, Arora P, Gross R, Seidel A, Hornich BF, Hahn AS, Kruger N, Graichen L, Hofmann-Winkler H, Kempf A, Winkler MS, Schulz S, Jack HM, Jahrsdorfer B, Schrezenmeier H, Muller M, Kleger A, Munch J, Pohlmann S. 2021. SARS-CoV-2 variants B.1.351 and P.1 escape from neutralizing antibodies. Cell 184:2384-2393 e12.

8. Jangra S, Ye C, Rathnasinghe R, Stadlbauer D, Personalized Virology Initiative study group, Krammer F, Simon V, Martinez-Sobrido L, Garcia-Sastre A, Schotsaert M. 2021. SARS-CoV-2 spike E484K mutation reduces antibody neutralisation. Lancet Microbe 2:e283-e284.

9. Garcia-Beltran WF, St Denis KJ, Hoelzemer A, Lam EC, Nitido AD, Sheehan ML, Berrios C, Ofoman O, Chang CC, Hauser BM, Feldman J, Roederer AL, Gregory DJ, Poznansky MC, Schmidt AG, Iafrate AJ, Naranbhai V, Balazs AB. 2022. mRNA-based COVID-19 vaccine boosters induce neutralizing immunity against SARS-CoV-2 Omicron variant. Cell doi:10.1016/j.cell.2021.12.033.

10. Liu Y, Liu J, Xia H, Zhang X, Fontes-Garfias CR, Swanson KA, Cai H, Sarkar R, Chen W, Cutler M, Cooper D, Weaver SC, Muik A, Sahin U, Jansen KU, Xie X, Dormitzer PR, Shi PY. 2021. Neutralizing activity of BNT162b2-elicited serum. N Engl J Med 384:1466-1468.

11. Liu Y, Liu J, Xia H, Zhang X, Zou J, Fontes-Garfias CR, Weaver SC, Swanson KA, Cai H, Sarkar R, Chen W, Cutler M, Cooper D, Muik A, Sahin U, Jansen KU, Xie X, Dormitzer PR, Shi PY. 2021. BNT162b2-elicited neutralization against new SARS-CoV-2 spike variants. N Engl J Med 385:472-474.

12. Liu J, Liu Y, Xia H, Zou J, Weaver SC, Swanson KA, Cai H, Cutler M, Cooper D, Muik A, Jansen KU, Sahin U, Xie X, Dormitzer PR, Shi PY. 2021. BNT162b2-elicited neutralization of B.1.617 and other SARS-CoV-2 variants. Nature 596:273-275.

13. Laurie MT, Liu J, Sunshine S, Peng J, Black D, Mitchell AM, Mann SA, Pilarowski G, Zorn KC, Rubio L, Bravo S, Marquez C, Petersen M, Havlir D, DeRisi J. 2021. Exposures to different SARS-CoV-2 spike variants elicit neutralizing antibody responses with differential specificity towards established and emerging strains. medRxiv doi:10.1101/2021.09.08.21263095:2021.09.08.21263095.

14. Messali S, Bertelli A, Campisi G, Zani A, Ciccozzi M, Caruso A, Caccuri F. 2021. A cluster of the new SARS-CoV-2 B.1.621 lineage in Italy and sensitivity of the viral isolate to the BNT162b2 vaccine. J Med Virol doi:10.1002/jmv.27247.

15. Muik A, Wallisch AK, Sanger B, Swanson KA, Muhl J, Chen W, Cai H, Maurus D, Sarkar R, Tureci O, Dormitzer PR, Sahin U. 2021. Neutralization of SARS-CoV-2 lineage B.1.1.7 pseudovirus by BNT162b2 vaccine-elicited human sera. Science 371:1152-1153.

16. Muik A, Lui BG, Wallisch A-K, Bacher M, Mühl J, Reinholz J, Ozhelvaci O, Beckmann N, Caridad Güimil Garcia Rdl, Poran A, Shpyro S, Cai H, Yang Q, Swanson KA, Türeci Ö, Sahin U. 2021. Neutralization of SARS-CoV-2 Omicron pseudovirus by BNT162b2 vaccine-elicited human sera. medRxiv doi:10.1101/2021.12.22.21268103:2021.12.22.21268103.

17. Parry HM, Tut G, Faustini S, Stephens C, Saunders P, Bentley C, Hilyard K, Brown K, Amirthalingam G, Charlton S, Leung S, Chiplin E, Coombes NS, Bewley KR, Penn EJ, Rowe C, Otter A, Watts R, D’Arcangelo S, Hallis B, Makin A, Richter AG, Zuo J. 2021. BNT162b2 vaccination in people over 80 years of age induces strong humoral immune responses with cross neutralisation of P.1 Brazilian variant. <https://ssrn.com/abstract=3816840>. Accessed 14 June 2021,

18. Peiris M, Cheng S, Ka Pun Mok C, Leung Y, Ng S, Chan K, Ko F, Yiu K, Lam B, Lau E, Chan K, Luk L, Li J, Tsang L, Poon L, Chen C, Hui D. 2022. Neutralizing antibody titres to SARS-CoV-2 Omicron variant and wild-type virus in those with past infection or vaccinated or boosted with mRNA BNT162b2 or inactivated CoronaVac vaccines. Nature Portfolio doi:10.21203/rs.3.rs-1207071/v1.

19. Planas D, Bruel T, Grzelak L, Guivel-Benhassine F, Staropoli I, Porrot F, Planchais C, Buchrieser J, Rajah MM, Bishop E, Albert M, Donati F, Prot M, Behillil S, Enouf V, Maquart M, Smati-Lafarge M, Varon E, Schortgen F, Yahyaoui L, Gonzalez M, De Seze J, Pere H, Veyer D, Seve A, Simon-Loriere E, Fafi-Kremer S, Stefic K, Mouquet H, Hocqueloux L, van der Werf S, Prazuck T, Schwartz O. 2021. Sensitivity of infectious SARS-CoV-2 B.1.1.7 and B.1.351 variants to neutralizing antibodies. Nat Med 27:917-924.

20. Planas D, Veyer D, Baidaliuk A, Staropoli I, Guivel-Benhassine F, Rajah MM, Planchais C, Porrot F, Robillard N, Puech J, Prot M, Gallais F, Gantner P, Velay A, Le Guen J, Kassis-Chikhani N, Edriss D, Belec L, Seve A, Courtellemont L, Pere H, Hocqueloux L, Fafi-Kremer S, Prazuck T, Mouquet H, Bruel T, Simon-Loriere E, Rey FA, Schwartz O. 2021. Reduced sensitivity of SARS-CoV-2 variant Delta to antibody neutralization. Nature 596:276-280.

21. Rössler A, Riepler L, Bante D, Laer Dv, Kimpel J. 2021. SARS-CoV-2 B.1.1.529 variant (Omicron) evades neutralization by sera from vaccinated and convalescent individuals. medRxiv doi:10.1101/2021.12.08.21267491:2021.12.08.21267491.

22. Skelly DT, Harding AC, Gilbert-Jaramillo J, Knight ML, Longet S, Brown A, Adele S, Adland E, Brown H, Tipton T, Stafford L, Mentzer AJ, Johnson SA, Amini A, Tan TK, Schimanski L, Huang KA, Rijal P, Frater J, Goulder P, Conlon CP, Jeffery K, Dold C, Pollard AJ, Sigal A, de Oliveira T, Townsend AR, Klenerman P, Dunachie SJ, Barnes E, Carroll MW, James WS. 2021. Two doses of SARS-CoV-2 vaccination induce robust immune responses to emerging SARS-CoV-2 variants of concern. Nat Commun 12:5061.

23. Supasa P, Zhou D, Dejnirattisai W, Liu C, Mentzer AJ, Ginn HM, Zhao Y, Duyvesteyn HME, Nutalai R, Tuekprakhon A, Wang B, Paesen GC, Slon-Campos J, Lopez-Camacho C, Hallis B, Coombes N, Bewley KR, Charlton S, Walter TS, Barnes E, Dunachie SJ, Skelly D, Lumley SF, Baker N, Shaik I, Humphries HE, Godwin K, Gent N, Sienkiewicz A, Dold C, Levin R, Dong T, Pollard AJ, Knight JC, Klenerman P, Crook D, Lambe T, Clutterbuck E, Bibi S, Flaxman A, Bittaye M, Belij-Rammerstorfer S, Gilbert S, Hall DR, Williams MA, Paterson NG, James W, Carroll MW, Fry EE, Mongkolsapaya J, Ren J, Stuart DI, Screaton GR. 2021. Reduced neutralization of SARS-CoV-2 B.1.1.7 variant by convalescent and vaccine sera. Cell 184:2201-2211 e7.

24. Tada T, Dcosta BM, Samanovic MI, Herati RS, Cornelius A, Zhou H, Vaill A, Kazmierski W, Mulligan MJ, Landau NR. 2021. Convalescent-phase sera and vaccine-elicited antibodies largely maintain neutralizing titer against global SARS-CoV-2 variant spikes. mBio 12:e0069621.

25. Uriu K, Kimura I, Shirakawa K, Takaori-Kondo A, Nakada T-a, Kaneda A, Nakagawa S, Sato K. 2021. Ineffective neutralization of the SARS-CoV-2 Mu variant by convalescent and vaccine sera. bioRxiv doi:10.1101/2021.09.06.459005:2021.09.06.459005.

26. Wall EC, Wu M, Harvey R, Kelly G, Warchal S, Sawyer C, Daniels R, Hobson P, Hatipoglu E, Ngai Y, Hussain S, Nicod J, Goldstone R, Ambrose K, Hindmarsh S, Beale R, Riddell A, Gamblin S, Howell M, Kassiotis G, Libri V, Williams B, Swanton C, Gandhi S, Bauer DL. 2021. Neutralising antibody activity against SARS-CoV-2 VOCs B.1.617.2 and B.1.351 by BNT162b2 vaccination. Lancet 397:2331-2333.

27. Wang P, Nair MS, Liu L, Iketani S, Luo Y, Guo Y, Wang M, Yu J, Zhang B, Kwong PD, Graham BS, Mascola JR, Chang JY, Yin MT, Sobieszczyk M, Kyratsous CA, Shapiro L, Sheng Z, Huang Y, Ho DD. 2021. Antibody resistance of SARS-CoV-2 variants B.1.351 and B.1.1.7. Nature 593:130-135.

28. Wilhelm A, Widera M, Grikscheit K, Toptan T, Schenk B, Pallas C, Metzler M, Kohmer N, Hoehl S, Helfritz FA, Wolf T, Goetsch U, Ciesek S. 2021. Reduced Neutralization of SARS-CoV-2 Omicron Variant by Vaccine Sera and Monoclonal Antibodies. medRxiv doi:10.1101/2021.12.07.21267432:2021.12.07.21267432.

29. Xie X, Zou J, Fontes-Garfias CR, Xia H, Swanson KA, Cutler M, Cooper D, Menachery VD, Weaver S, Dormitzer PR, Shi PY. 2021. Neutralization of N501Y mutant SARS-CoV-2 by BNT162b2 vaccine-elicited sera. bioRxiv doi:10.1101/2021.01.07.425740.

30. Xie X, Liu Y, Liu J, Zhang X, Zou J, Fontes-Garfias CR, Xia H, Swanson KA, Cutler M, Cooper D, Menachery VD, Weaver S, Dormitzer PR, Shi PY. 2021. Neutralization of SARS-CoV-2 spike 69/70 deletion, E484K, and N501Y variants by BNT162b2 vaccine-elicited sera. bioRxiv doi:10.1101/2021.01.27.427998.

31. Doria-Rose NA, Shen X, Schmidt SD, O’Dell S, McDanal C, Feng W, Tong J, Eaton A, Maglinao M, Tang H, Manning KE, Edara V-V, Lai L, Ellis M, Moore K, Floyd K, Foster SL, Atmar RL, Lyke KE, Zhou T, Wang L, Zhang Y, Gaudinski MR, Black WP, Gordon I, Guech M, Ledgerwood JE, Misasi JN, Widge A, Roberts PC, Beigel J, Korber B, Pajon R, Mascola JR, Suthar MS, Montefiori DC. 2021. Booster of mRNA-1273 Strengthens SARS-CoV-2 Omicron Neutralization. medRxiv doi:10.1101/2021.12.15.21267805:2021.12.15.21267805.

32. Edara VV, Norwood C, Floyd K, Lai L, Davis-Gardner ME, Hudson WH, Mantus G, Nyhoff LE, Adelman MW, Fineman R, Patel S, Byram R, Gomes DN, Michael G, Abdullahi H, Beydoun N, Panganiban B, McNair N, Hellmeister K, Pitts J, Winters J, Kleinhenz J, Usher J, O'Keefe JB, Piantadosi A, Waggoner JJ, Babiker A, Stephens DS, Anderson EJ, Edupuganti S, Rouphael N, Ahmed R, Wrammert J, Suthar MS. 2021. Infection- and vaccine-induced antibody binding and neutralization of the B.1.351 SARS-CoV-2 variant. Cell Host Microbe 29:516-521 e3.

33. Edara VV, Hudson WH, Xie X, Ahmed R, Suthar MS. 2021. Neutralizing antibodies against SARS-CoV-2 variants after infection and vaccination. JAMA 325:1896-1898.

34. Shen X, Tang H, McDanal C, Wagh K, Fischer W, Theiler J, Yoon H, Li D, Haynes BF, Sanders KO, Gnanakaran S, Hengartner N, Pajon R, Smith G, Glenn GM, Korber B, Montefiori DC. 2021. SARS-CoV-2 variant B.1.1.7 is susceptible to neutralizing antibodies elicited by ancestral spike vaccines. Cell Host Microbe 29:529-539 e3.

35. Wu K, Werner AP, Koch M, Choi A, Narayanan E, Stewart-Jones GBE, Colpitts T, Bennett H, Boyoglu-Barnum S, Shi W, Moliva JI, Sullivan NJ, Graham BS, Carfi A, Corbett KS, Seder RA, Edwards DK. 2021. Serum Neutralizing Activity Elicited by mRNA-1273 Vaccine. N Engl J Med 384:1468-1470.

36. Wu K, Werner AP, Moliva JI, Koch M, Choi A, Stewart-Jones GBE, Bennett H, Boyoglu-Barnum S, Shi W, Graham BS, Carfi A, Corbett KS, Seder RA, Edwards DK. 2021. mRNA-1273 vaccine induces neutralizing antibodies against spike mutants from global SARS-CoV-2 variants. bioRxiv doi:10.1101/2021.01.25.427948:2021.01.25.427948.

37. Schmidt F, Muecksch F, Weisblum Y, Silva JD, Bednarski E, Cho A, Wang Z, Gaebler C, Caskey M, Nussenzweig MC, Hatziioannou T, Bieniasz PD. 2021. Plasma neutralization properties of the SARS-CoV-2 Omicron variant. medRxiv doi:10.1101/2021.12.12.21267646:2021.12.12.21267646.

38. Wang P, Casner RG, Nair MS, Wang M, Yu J, Cerutti G, Liu L, Kwong PD, Huang Y, Shapiro L, Ho DD. 2021. Increased resistance of SARS-CoV-2 variant P.1 to antibody neutralization. Cell Host Microbe 29:747-751 e4.

39. Wang Z, Schmidt F, Weisblum Y, Muecksch F, Barnes CO, Finkin S, Schaefer-Babajew D, Cipolla M, Gaebler C, Lieberman JA, Oliveira TY, Yang Z, Abernathy ME, Huey-Tubman KE, Hurley A, Turroja M, West KA, Gordon K, Millard KG, Ramos V, Da Silva J, Xu J, Colbert RA, Patel R, Dizon J, Unson-O'Brien C, Shimeliovich I, Gazumyan A, Caskey M, Bjorkman PJ, Casellas R, Hatziioannou T, Bieniasz PD, Nussenzweig MC. 2021. mRNA vaccine-elicited antibodies to SARS-CoV-2 and circulating variants. Nature 592:616-622.

40. Emary KRW, Golubchik T, Aley PK, Ariani CV, Angus B, Bibi S, Blane B, Bonsall D, Cicconi P, Charlton S, Clutterbuck EA, Collins AM, Cox T, Darton TC, Dold C, Douglas AD, Duncan CJA, Ewer KJ, Flaxman AL, Faust SN, Ferreira DM, Feng S, Finn A, Folegatti PM, Fuskova M, Galiza E, Goodman AL, Green CM, Green CA, Greenland M, Hallis B, Heath PT, Hay J, Hill HC, Jenkin D, Kerridge S, Lazarus R, Libri V, Lillie PJ, Ludden C, Marchevsky NG, Minassian AM, McGregor AC, Mujadidi YF, Phillips DJ, Plested E, Pollock KM, Robinson H, Smith A, Song R, Snape MD, Sutherland RK, Thomson EC, Toshner M, Turner DPJ, Vekemans J, Villafana TL, Williams CJ, Hill AVS, Lambe T, Gilbert SC, Voysey M, Ramasamy MN, Pollard AJ, consortium C-GU, Project A, Oxford C-VTG. 2021. Efficacy of ChAdOx1 nCoV-19 (AZD1222) vaccine against SARS-CoV-2 variant of concern 202012/01 (B.1.1.7): an exploratory analysis of a randomised controlled trial. Lancet 397:1351-1362.

41. Madhi SA, Baillie V, Cutland CL, Voysey M, Koen AL, Fairlie L, Padayachee SD, Dheda K, Barnabas SL, Bhorat QE, Briner C, Kwatra G, Ahmed K, Aley P, Bhikha S, Bhiman JN, Bhorat AE, du Plessis J, Esmail A, Groenewald M, Horne E, Hwa SH, Jose A, Lambe T, Laubscher M, Malahleha M, Masenya M, Masilela M, McKenzie S, Molapo K, Moultrie A, Oelofse S, Patel F, Pillay S, Rhead S, Rodel H, Rossouw L, Taoushanis C, Tegally H, Thombrayil A, van Eck S, Wibmer CK, Durham NM, Kelly EJ, Villafana TL, Gilbert S, Pollard AJ, de Oliveira T, Moore PL, Sigal A, Izu A, Group N-S, Wits VCG. 2021. Efficacy of the ChAdOx1 nCoV-19 Covid-19 vaccine against the B.1.351 variant. N Engl J Med 384:1885-1898.

42. Alter G, Yu J, Liu J, Chandrashekar A, Borducchi EN, Tostanoski LH, McMahan K, Jacob-Dolan C, Martinez DR, Chang A, Anioke T, Lifton M, Nkolola J, Stephenson KE, Atyeo C, Shin S, Fields P, Kaplan I, Robins H, Amanat F, Krammer F, Baric RS, Le Gars M, Sadoff J, de Groot AM, Heerwegh D, Struyf F, Douoguih M, van Hoof J, Schuitemaker H, Barouch DH. 2021. Immunogenicity of Ad26.COV2.S vaccine against SARS-CoV-2 variants in humans. Nature 596:268-272.

43. Moore PL, Moyo-Gwete T, Hermanus T, Kgagudi P, Ayres F, Makhado Z, Sadoff J, Le Gars M, van Roey G, Crowther C, Garrett N, Bekker L-G, Morris L, Schuitemaker H, Gray G. 2021. Neutralizing antibodies elicited by the Ad26.COV2.S COVID-19 vaccine show reduced activity against 501Y.V2 (B.1.351), despite protection against severe disease by this variant. bioRxiv doi:10.1101/2021.06.09.447722:2021.06.09.447722.

44. Ikegame S, Siddiquey MNA, Hung CT, Haas G, Brambilla L, Oguntuyo KY, Kowdle S, Chiu HP, Stevens CS, Vilardo AE, Edelstein A, Perandones C, Kamil JP, Lee B. 2021. Neutralizing activity of Sputnik V vaccine sera against SARS-CoV-2 variants. Nat Commun 12:4598.
