# Supplementary material for: The Impact of Evolving SARS-CoV-2 Mutations and Variants on COVID-19 Vaccines
Source: mBio. 2022 Mar 30;13(2):e02979-21. doi: 10.1128/mbio.02979-21 (PMC9040821; doi:10.1128/mbio.02979-21)
Supplement: TABLE S3 [file mbio.02979-21-s0003.docx]

Table S3 Efficacy of COVID-19 vaccines against SARS-CoV-2 variants (1-6)

| **Variant** | **Vaccine** | **Design and setting** | **Key outcomes** | **Vaccine efficacy, % (95% CI)** | **Reference** |
| --- | --- | --- | --- | --- | --- |
| Alpha | ChAdOx1-nCoV-19 | Phase 2/3 trial in the United Kingdom | PCR-confirmed symptomatic infection caused by alpha | 70.4 (43.6 to 84.5) | Emary KRW, et al. 2021 (1) |
|  |  |  | PCR-confirmed asymptomatic infection or unknown symptoms caused by alpha | 28.9 (−77.1 to 71.4) |  |
|  |  |  | All infections caused by alpha | 61.7 (36.7 to 76.9) |  |
|  | NVX-CoV2373 | Phase 3 trial in United Kingdom | PCR-confirmed symptomatic COVID-19 caused by alpha | 85.6 (CI not reported) | Novavax 2021 (2) |
| Beta | ChAdOx1-nCoV-19 | Phase 1b/2 trial in South Africa | PCR-confirmed symptomatic mild-to-moderate COVID-19 caused by beta | 10.4 (−76.8 to 54.8) | Madhi SA, et al. 2021 (3) |
|  | NVX-CoV2373 | Phase 2 trial in South Africa, participants without HIV, 38 of 41 events due to beta | PCR-confirmed symptomatic COVID-19 | 60.1 (19.9 to 80.1) | Shinde V, et al. 2021 (4) |
|  | Ad26.COV2.S | Phase 3 trial, South African cohort, 95% of events due to beta | PCR-confirmed moderate-to-severe COVID-19 | 57 (CI not reported) | Johnson & Johnson 2021 (5) |
|  | BNT162b2 | Phase 2/3 trial, South African cohort; 8 of 9 events due to beta, one lineage undetermined | PCR-confirmed symptomatic COVID-19 | 100 (53.5 to 100.0) | Thomas SJ, et al. 2021 (6) |
| Delta | BNT162b2 | Phase 3 trial in the United States, Brazil, and South Africa, delta prevalent strain | PCR-confirmed symptomatic COVID-19 | 95.6 (89.3 to 98.6) | BioNTech/Pfizer 2021 (7) |

CI, confidence interval; COVID-19, coronavirus disease 2019; HIV, human immunodeficiency virus; PCR, polymerase chain reaction; SARS-CoV-2, severe acute respiratory syndrome coronavirus 2.

# References

1. Emary KRW, Golubchik T, Aley PK, Ariani CV, Angus B, Bibi S, Blane B, Bonsall D, Cicconi P, Charlton S, Clutterbuck EA, Collins AM, Cox T, Darton TC, Dold C, Douglas AD, Duncan CJA, Ewer KJ, Flaxman AL, Faust SN, Ferreira DM, Feng S, Finn A, Folegatti PM, Fuskova M, Galiza E, Goodman AL, Green CM, Green CA, Greenland M, Hallis B, Heath PT, Hay J, Hill HC, Jenkin D, Kerridge S, Lazarus R, Libri V, Lillie PJ, Ludden C, Marchevsky NG, Minassian AM, McGregor AC, Mujadidi YF, Phillips DJ, Plested E, Pollock KM, Robinson H, Smith A, Song R, Snape MD, Sutherland RK, Thomson EC, Toshner M, Turner DPJ, Vekemans J, Villafana TL, Williams CJ, Hill AVS, Lambe T, Gilbert SC, Voysey M, Ramasamy MN, Pollard AJ, consortium C-GU, Project A, Oxford C-VTG. 2021. Efficacy of ChAdOx1 nCoV-19 (AZD1222) vaccine against SARS-CoV-2 variant of concern 202012/01 (B.1.1.7): an exploratory analysis of a randomised controlled trial. Lancet 397:1351-1362.

2. Novavax. 2021. Novavax COVID-19 Vaccine Demonstrates 89.3% Efficacy in UK Phase 3 Trial. <https://ir.novavax.com/news-releases/news-release-details/novavax-covid-19-vaccine-demonstrates-893-efficacy-uk-phase-3>. Accessed 03 March, 2021.

3. Madhi SA, Baillie V, Cutland CL, Voysey M, Koen AL, Fairlie L, Padayachee SD, Dheda K, Barnabas SL, Bhorat QE, Briner C, Kwatra G, Ahmed K, Aley P, Bhikha S, Bhiman JN, Bhorat AE, du Plessis J, Esmail A, Groenewald M, Horne E, Hwa SH, Jose A, Lambe T, Laubscher M, Malahleha M, Masenya M, Masilela M, McKenzie S, Molapo K, Moultrie A, Oelofse S, Patel F, Pillay S, Rhead S, Rodel H, Rossouw L, Taoushanis C, Tegally H, Thombrayil A, van Eck S, Wibmer CK, Durham NM, Kelly EJ, Villafana TL, Gilbert S, Pollard AJ, de Oliveira T, Moore PL, Sigal A, Izu A, Group N-S, Wits VCG. 2021. Efficacy of the ChAdOx1 nCoV-19 Covid-19 vaccine against the B.1.351 variant. N Engl J Med 384:1885-1898.

4. Shinde V, Bhikha S, Hoosain Z, Archary M, Bhorat Q, Fairlie L, Lalloo U, Masilela MSL, Moodley D, Hanley S, Fouche L, Louw C, Tameris M, Singh N, Goga A, Dheda K, Grobbelaar C, Kruger G, Carrim-Ganey N, Baillie V, de Oliveira T, Lombard Koen A, Lombaard JJ, Mngqibisa R, Bhorat AE, Benade G, Lalloo N, Pitsi A, Vollgraaff PL, Luabeya A, Esmail A, Petrick FG, Oommen-Jose A, Foulkes S, Ahmed K, Thombrayil A, Fries L, Cloney-Clark S, Zhu M, Bennett C, Albert G, Faust E, Plested JS, Robertson A, Neal S, Cho I, Glenn GM, Dubovsky F, Madhi SA, 2019nCoV-501 Study Group. 2021. Efficacy of NVX-CoV2373 Covid-19 vaccine against the B.1.351 variant. N Engl J Med 384:1899-1909.

5. Johnson & Johnson. 2021. Johnson & Johnson Announces Single-Shot Janssen COVID-19 Vaccine Candidate Met Primary Endpoints in Interim Analysis of its Phase 3 ENSEMBLE Trial. <https://www.jnj.com/johnson-and-johnson-announces-single-shot-janssen-covid-19-vaccine-candidate-met-primary-endpoints-in-interim-analysis-of-its-phase-3-ensemble-trial>. Accessed 03 March, 2021.

6. Thomas SJ, Moreira ED, Kitchin N, Absalon J, Gurtman A, Lockhart S, Perez JL, Pérez Marc G, Polack FP, Zerbini C, Bailey R, Swanson KA, Xu X, Roychoudhury S, Koury K, Bouguermouh S, Kalina WV, Cooper D, Frenck RW, Hammitt LL, Türeci Ö, Nell H, Schaefer A, Ünal S, Yang Q, Liberator P, Tresnan DB, Mather S, Dormitzer PR, Şahin U, Gruber WC, Jansen KU. 2021. Six month safety and efficacy of the BNT162b2 mRNA COVID-19 vaccine. medRxiv doi:10.1101/2021.07.28.21261159:2021.07.28.21261159.

7. Pfizer, BioNTech. 2021. Pfizer and BioNTech Announce Phase 3 Trial Data Showing High Efficacy of a Booster Dose of Their COVID-19 Vaccine.
